# Do you reap what you sow? Driving mechanism of supply chain transparency on consumers' indirect reciprocity

**DOI:** 10.3389/fpsyg.2023.1081297

**Published:** 2023-02-09

**Authors:** Shaohua He

**Affiliations:** School of Economics and Management, South China Agricultural University, Guangzhou, China

**Keywords:** trust, indirect reciprocity, alleviation poverty program, supply chain transparency, personality heterogeneity

## Abstract

**Introduction:**

To maintain sustainable poverty alleviation in the post-pandemic world, China encourages firms to participate in the “Social Commerce Helping Farmers Project.” This study aims to explore the phenomenon of indirect reciprocity between firms, consumers, and farmers in the supply chain. This study explores how supply chain transparency stimulates indirect reciprocity among consumers through competence trust, goodwill trust, and integrity trust. Furthermore, we explore the impact of compassion and the need for social status on the model.

**Methods:**

We fit a partial least square analysis structural equation modeling (PLS-SEM) using data from an online random vignette-based experiment questionnaire survey.

**Results:**

Supply chain transparency of social responsibility practices asymmetrically affects three dimensions of consumer trust by improving perceived information quality. And the three dimensions of trust asymmetrically contribute to indirect reciprocity. Furthermore, compassion has a positive moderating effect on the relationship between perceived information quality and trust. However, the moderating effect of the need for social status on the relationship between the three dimensions of trust and indirect reciprocity differed significantly.

**Discussion:**

Our findings indicate that supply chain transparency improves consumer trust, making consumers more responsive and rewarding companies that assist vulnerable groups in their supply chains. Faced with a trust crisis, companies can take different measures to achieve their goals based on each dimension of trust. At the same time, companies need to consider differences in the responses of consumers with different personality traits (e.g., compassion and the need for social status) when revealing their corporate social responsibility practices to consumers.

## Introduction

Ending poverty in all its forms everywhere is the first goal of the 2030 Sustainable Development Goals. Raising farmers' incomes is central to anti-poverty efforts because 80% the poor in developing countries live in rural areas (World Social Report, 2021). In the Millennium Declaration, the United Nations encouraged remote poor areas to make full use of information technology to escape poverty. Goh (2022) shows that e-commerce, as an application field of information technology, can effectively play the role of helping farmers by combining consumers and enterprises.

Since 2015, Combined with the social commerce era background, China has piloted the “Social Commerce Helping Farmer” project. Especially since the outbreak of COVID-19, enterprises on major S-commerce platforms, encouraged by the state and expected by society, have launched “helping farmers” projects, including agricultural products from poor areas or areas seriously affected by the epidemic into the procurement end of raw materials, and registered to help raw material producers. Social commerce functions on e-commerce websites establish a bridge of communication between enterprises and consumers (Huang and Benyoucef, 2013). The rise of social commerce has provided a platform for companies to provide transparent supply chain information to consumers. This offers the possibility for consumers to engage with and respond to the actions of companies. It also offers the possibility of sustainable operation of the project.

To be more specific, The core of achieving project objectives is that the market can drive the implementation of the project spontaneously, which means that the “helping farmer” behavior of firms arouses the positive response or indirect reciprocity of consumers toward firms. The participation of enterprises in helping farmers is a kind of corporate social responsibility practice or prosocial behavior. It is also a prosocial motivation when consumers engage in indirect reciprocity. This intention to promote the well-being of others is considered other-oriented prosocial behavior (Carlo, 2006; Pfattheicher et al., 2022). There is evidence to suggest that consumers respond to efforts of companies to promote their corporate social responsibility activities (Han et al., 2019; Zhuang et al., 2021).

The indirect reciprocity of consumers has served as an “enabling” factor, allowing the initiative to continue. Indirect reciprocity is the main mechanism fostering assistance or generosity between three unfamiliar subjects (Romano et al., 2022). Indirect reciprocity is proper to explain consumer reactions to “the ethical corporate conduct of assisting vulnerable supply chain groups.” Following Engelmann and Fischbacher (2009), *indirect reciprocity* is when a third party rewards and punishes one party for friendly or hostile behavior against another. Therefore, indirect reciprocity occurs when individuals help those who have assisted others. This study is based on the following social scenarios: On the social commerce platform, The firm declares its participation in the “social commerce agriculture project.” After viewing the information, consumers will initiate the process of indirect reciprocity. Our research can complement research on consumer motivation from the perspective of indirect reciprocity.

Indirect reciprocity can predict whether consumers are willing to pay costs to reward firms, which is a crucial issue in business management and marketing research (Jaeger and Weber, 2020; Diallo et al., 2021; Yu et al., 2022). When indirect reciprocity is unlikely, firms will stop investing in pro-social behavior (Simpson and Willer, 2008). Enterprises wish to obtain more evident consumer attitudes toward CSR practices (Mohr et al., 2001; Green and Peloza, 2011; Sodhi and Tang, 2019). Sodhi and Tang (2019) points out that it is costly to collect and disclose information in the supply chain on websites, and firms need to be clear about consumer responses to provide supply chain transparency.

Particularly in developing countries, there needs to be further research on public responses to specific CSR programs in supply chains (Idemudia, 2011; Ackers, 2015). Increasing numbers of firms choose to collect and disclose this information (Marshall et al., 2016), but the benefits of such transparency have yet to be determined (Sodhi and Tang, 2019). Companies are discouraged from implementing CSR programs due to a lack of understanding of consumer descriptions of specific activities (Bhattacharya and Sen, 2004; Sen et al., 2016). For these firms, indirect reciprocity was a significant predictor. Charness and Rabin (2002) and Danz et al. (2022) demonstrates that consumers' strategy of considering the interests of others can be captured by indirect reciprocity.

Trust is essential for generating fairness, care, and other-oriented concern (Mishler, 2002), which can stimulate other-directed prosocial behavior (Deutsch, 1958). Trust acts as a mediator in consumer research to influence consumers' positive perceptions and attitudes (Fang et al., 2014; Taheri and Shourmasti, 2016; Kamboj et al., 2018; Liang et al., 2018; Song et al., 2019; Xiao et al., 2019; Iglesias et al., 2020). Thus, trust is seen as the antecedent variable of consumers' indirect reciprocity.

In the e-commerce transaction environment, trust remains a challenge (Hajli, 2019). Consumers cannot easily believe the company's simple statement because of the suspicion that the company may manipulate “ethical products” (Vanhamme and Grobben, 2009; Skarmeas and Leonidou, 2013). This skepticism may hinder the positive perception of the firm by consumers, as well as the behavior that motivates good business (Skarmeas and Leonidou, 2013).

In the face of doubt, one of the solutions is increasing the transparency of a sustainable corporate social responsibility supply chain (Marshall et al., 2016). For instance, Clarke et al. (2007) studies on ethical consumption campaigns in the United Kingdom reveal that these campaigns emphasize providing people with information to support certain causes and extend their care and responsibility to daily consumption practices. Existing studies have examined the relationship between supply chain transparency and consumer trust (Xiao et al., 2019), but there is still a need for additional research on the three dimensions of trust (goodwill, competence, and integrity). The three dimensions of consumer trust may play different roles in different social contexts (Wu et al., 2021). Further, we intend to investigate the relationship between the three dimensions of trust and indirect reciprocity.

Capturing consumer heterogeneity in decision-making plays a vital role (Shim, 2021). Compassion and the need for social status are the main driving forces for individuals to make decisions that benefit others (Goetz et al., 2010; Griskevicius et al., 2010; Lim and DeSteno, 2016; Khan and Fazili, 2019). Previous research has shown that individual differences in compassion and the need for social status influence consumers' prosocial behaviors (Grier and Deshpandé, 2001; Condon and DeSteno, 2011; Puska et al., 2018; de Morais et al., 2021). During the indirect reciprocity decision-making process, consumers' compassion preferences may resonate with the information provided, and the preference for social status needs may drive consumers to take action. Therefore, considering the effect of compassion and the need for social status preferences on consumers' indirect reciprocity processes will be more in line with realistic decision-making situations.

In the context of China's “Social Commerce Helping Farmers,” we derive and verify an indirect reciprocity process theory framework to explore the following questions: 1. the relationship between supply chain transparency considering social responsibility and consumer trust in businesses; 2. the impact of supply chain transparency on consumer indirect reciprocity; 3. the role played by consumer trust in the relationships described above; and 4. the impact of consumer heterogeneity (compassion, need for social status) on the model.

The contribution of this study is fivefold: 1. presents a theoretical framework for China's poverty alleviation project, “Social Commerce Helping Farmers.”; 2. complements research that considers supply chain transparency in socially responsible practices; 3. supplements the study of the three dimensions of trust as an intermediary mechanism; 4. constructs and validates a theoretical framework for indirect reciprocity in consumer decision-making research; 5. explores the boundaries of indirect reciprocity theory: compassion and the need for social status.

## Theoretical background

In response to national policies and consumer expectations, social commerce platforms and merchants on the platform began to carry out the “Social Commerce Helping Farmers” project according to their product characteristics. Specifically, some companies have started to show on their product web pages that they are supporting poor growers of raw materials in the supply chain. However, the information displayed on the web was distinct. Some companies show exactly how and where in the supply chain they help farmers, while others simply attach a few words near the product name to claim that they participate in programs that help farmers. Using such a design, customers can learn about the social practices of enterprises. We have this intuition that it's critical to see the process by which companies help farmers, or that supply chain transparency is key.

The foundation of the project lies in the rise of social commerce. As social media and Web 2.0 have rapidly developed, e-commerce has evolved from a product-centered environment to one that is customer-centered (Huang and Benyoucef, 2013). In this context, customers can make better decisions online by using transparent information (Busalim et al., 2016). Customers' positive responses will enable companies participating in the “Social Commerce Helping Farmers” program to do more to assist farmers.

In 1987, indirect reciprocity theory was first proposed by Alexander to explain the moral system of biology (Alexander, 2017). As the origin of human ethics, indirect reciprocity facilitates the spreading of kindness among strangers. It evoked from the fields of anthropology and psychology to economics, sociology, and management (Boyd and Richerson, 1989; Bateson et al., 2006; Baker and Bulkley, 2014; Bock et al., 2021).

Indirect reciprocity can be distinguished into upstream indirect reciprocity and downstream indirect reciprocity based on the direction of transmission of kindness (Nowak and Roch, 2007). In upstream reciprocity, B first accepts the kindness of A and then transmits it to C (Bartlett and DeSteno, 2006). Downstream indirect reciprocity is that B releases generosity to C first, and after A observes this generosity, A inspires kindness or rewards to B (Nowak and Sigmund, 1998; Chalub et al., 2006; Szcześniak et al., 2020). Upstream indirect reciprocity facilitates the virtuous circle of generosity, whereas downstream indirect reciprocity does not (Nowak and Roch, 2007).

Downstream indirect reciprocity is constantly proliferating and evolving, and he focuses on the third party's behavior (O) (Roberts, 2008). The observer (O) first observes B's behavior toward A and then chooses how to treat B (Boyd and Richerson, 1989; Nowak and Sigmund, 2005). It explains the phenomenon that people pay a monetary cost to benefit unrelated strangers (Nowak and Sigmund, 2005; Thielmann et al., 2021; van Dijk and De Dreu, 2021).

Downstream indirect reciprocity decisions rely on the clues about the partner (Roberts et al., 2021). An indirect reciprocity process begins with the observation information provided by B (Wedekind and Milinski, 2000; Fehr and Fischbacher, 2003; Rockenbach and Milinski, 2006; Sommerfeld et al., 2007). Proactive and effective communication with third parties can benefit B (Nowak and Sigmund, 1998, 2005; Brandt and Sigmund, 2006; Ohtsuki and Iwasa, 2006).

By displaying information about how their supply chain benefits farmers, enterprises are able to encourage consumers to make indirect reciprocal decisions. Through social commerce platforms, enterprises can make information transparent to consumers (Huang and Benyoucef, 2013). It is vital that information be conveyed effectively (Sommerfeld et al., 2007; Rand and Nowak, 2013; Suzuki and Kimura, 2013). That is to say, “the clues provided by companies to help farmers” (supply chain transparency) must be perceived by consumers (perception of information quality).

Consumers' perceptions of information will translate into “judgments of cues” (trust) that stimulate indirect reciprocity intentions. Transparency of other people's interest-oriented leads in online systems can affect customer trust (Bock et al., 2021). Establishing a credible system will bring economic value to the enterprise (Resnick et al., 2006). Transparent and credible information can facilitate effective indirect reciprocity (Schmid et al., 2021).

For consumers, indirect reciprocity is a prosocial decision process in the context of social commerce helping farmers. Since compassion and the need for social status are intrinsic drivers of prosocial decision-making (Grier and Deshpandé, 2001; Condon and DeSteno, 2011; Puska et al., 2018; de Morais et al., 2021), compassion (Trivers, 1971) and the need for social status (Seinen and Schram, 2006) may moderate this process. *Compassion* is a progressive emotion that refers to feeling sorry for the pain of others and arousing a desire to help others (Goetz et al., 2010). It affects the tendency of individuals to behave prosocially (Saslow et al., 2013). It affects trust, but in what direction is debated (Liu and Wang, 2010; Spikins, 2015; Lupoli et al., 2020; Nathoo et al., 2021).

The need for social status refers to a primary human motivation for societal recognition and prestige (Eastman et al., 1999; Dubois et al., 2012). The level of need for social status affects people's prosocial behavior decisions (Blader and Chen, 2011). But there is some controversy about the direction of the impact (Stamos et al., 2020). Some studies suggest that the direction is positive: those with a higher need for social status are more motivated to help others (Flynn et al., 2006; Hardy and Van Vugt, 2006) and, the need for social status is one of the drivers of consumer involvement in prosocial activities (Pinto et al., 2019). Falk and Zehnder (2007), Korndörfer et al. (2015), and Silva and Mace (2015) also indicates that people from districts with high social status show more reporting in the trust game than people with low social status. However, some studies have come to the opposite conclusion: that people with a higher need for social status may be less likely to exhibit pro-social or perhaps even anti-social tendencies (Li and Wright, 2014; Guinote et al., 2015). Piff et al. (2010), Piff and Robinson (2017), and Amir et al. (2018) points out that individuals from lower social status are more sensitive to the welfare of others and more likely to show generosity in the trust game than those from higher ones.

The effect of the need for social status on pro-social tendencies may be relevant depending on the context or the particular type of pro-social behavior (Kafashan et al., 2014). We will explore these paradoxes through differences in the performance of people with different needs for social status in the relationship between trust (goodwill, competence, and integrity) and indirect reciprocity. According to the relevant research (Tajfel et al., 1986; Zahavi, 1995; Tajfel and Turner, 2004), it affects people's final intentions toward the products of prosocial attributes; therefore, it may act as a situational mechanism to influence the relationship between trust and indirect reciprocity.

**Figure 1:**
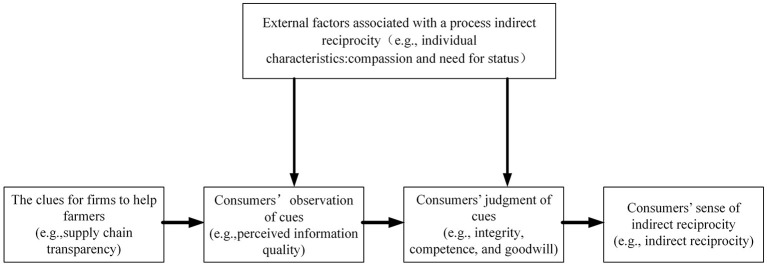
Indirect reciprocity process.

Based on theory and realistic circumstances, we developed the following framework (see Figure 1). Merchants on social commerce platforms generously assist poor farmers in the supply chain (C) and then disclose the information to consumers (A). This process is captured by supply chain transparency. Consumers (C) then evaluate and judge the observed information. This process involves perceived information quality and trust. Ultimately, consumers will likely have positive intentions or rewards for merchants (B). This intention is portrayed through indirect reciprocity.

We assume that trust is a prerequisite for indirect reciprocity in S-commerce. Similarly, some researchers in the field of philanthropy view trust as a prerequisite for philanthropic giving. They contend that if donors are to be effective altruists, they need to believe in an organization's ability to provide tangible benefits to its beneficiaries (Gaskin, 1999; Bekkers, 2006; Hager and Hedberg, 2016; Becker, 2018). Xu and Zhang (2022) used a Chinese national survey and found that institutional trust significantly predicted people's donations and volunteer activities. Moreover, it has been suggested that trust is one of the most critical determinants of prosocial behavior (Penner et al., 2005; Bear and Rand, 2016; Rand, 2016).

## Research model and hypotheses development

This section develops several research hypotheses. Based on indirect reciprocity theory, we operationalize: “the clues for businesses to help farmers” as supply chain transparency; “consumers' observations of cues” as perceived information quality; “consumers' judgment of cues” as integrity, competence, and goodwill; “consumers' sense of indirect reciprocity” as indirect reciprocity; and “external factors associated with indirect reciprocal decisions” as compassion and the need for social status.

**Figure 2:**
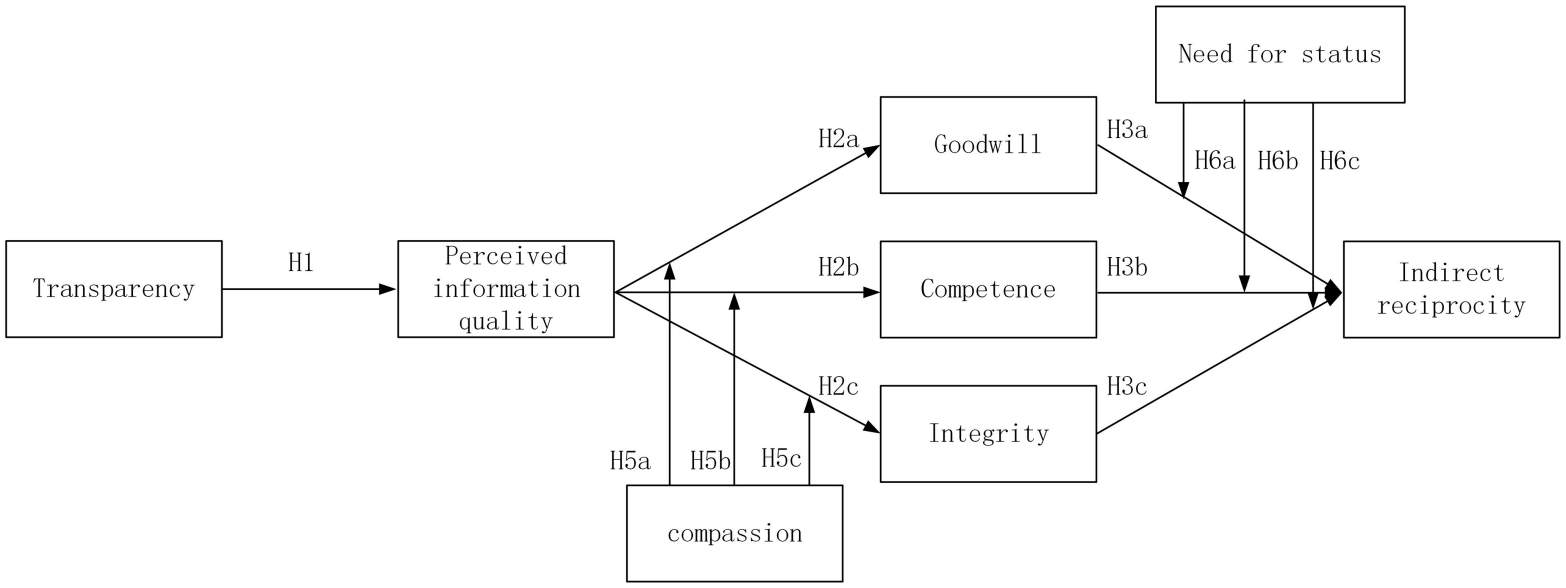
The theory framework and hypothesis. H4a, H4b, and H4c is the mediating effect hypothesis.

We propose that supply chain transparency can transfer from perceived information quality to trusting beliefs, forming indirect reciprocal intentions. Compassion and the need for social status may moderate the indirect reciprocity process. Compassion may enhance the transfer of perceived information quality to trust beliefs (integrity, competence, and goodwill). The need for social status may intensify the transfer of trust beliefs (integrity, competence, and goodwill) to indirect reciprocity. Figure 2 presents the research model and hypotheses.

### Supply chain transparency and perceived information quality

The network trading system between organizations enables enterprises to realize transparent transactions. In the previous supply chain management model, companies gained value by controlling manufacturing quality and inter-organizational costs. Transparency in supplier trading systems means providing multiple quantities and types of information to facilitate stakeholder determination of whether to trade (Finel and Lord, 1999). Its focus is on cost information in transactions, which is mainly transparent to supply-side partners or corporate shareholders (Williams, 2005). For example, Nicolaou and McKnight (2006) explores the role of information quality in the success of data exchange between organizations and points out that the trading system's transparency will affect the counterparties' perception of information quality.

The emergence of social commerce provides a channel for transparent communication between enterprises and consumers. With the transformation of scarce resources from products to consumers, enterprise information sharing aims to meet consumers' information needs. Current research focuses on disclosing the information consumers expect to receive in the supply chain (Zhou et al., 2018; Busser and Shulga, 2019).

Supply chain transparency has been redefined as companies disclosing information in the supply chain that consumers, investors, and other stakeholders need (Sodhi and Tang, 2019). Consumers want to encourage companies to do good through their decision-making behavior (Gómez-Corona, 2020). For example, Consumers expect organizations to behave socially responsibly, and they care about whether companies engage in specific practices (e.g., promoting minority employees, whether they hire children, whether they care about local schools) (Harrison et al., 2005). Additionally, consumers want to know as much as possible about CSR practices (Podnar, 2008) as it is challenging to determine whether companies are operating per their social responsibility standards.

The level of supply chain transparency affects consumers' perception of information quality (PIQ) (Nicolaou and McKnight, 2006; Chan et al., 2021). To achieve transparency, companies disclose precise information about supply chain operations and products, for example, the manufacturing processes and sustainability conditions of their suppliers (Egels-Zandén et al., 2015; Bai and Sarkis, 2020). PIQ is the consumer's perception of the accuracy, reliability, and utility of information (Yang, 2021). Therefore, we propose the following:

**Hypothesis 1**. Supply chain transparency positively affects perceived information quality.

### Perceived information quality and trust beliefs

In social commerce, the interaction between firms and consumers creates consumer value by integrating resources (Hajli et al., 2017). Firms achieve this interaction by making CSR practice information transparent to consumers. This value realization aims to reduce consumers' uncertainty or information asymmetry between merchants and consumers so that consumers can make favorable decisions (Kanani and Glavee-Geo, 2021). For example, In a study of users' willingness to exchange data, information quality translates into trust, then affects consumers' intention (Nicolaou et al., 2013).

Since trust is a psychological perception factor, it can be categorized into different dimensions according to the characteristics of trust objects. For example, Wongkitrungrueng and Assarut (2020) divides trust into cognitive trust and affects trust when studying customers' perceived value of live streaming. Nicolaou et al. (2013) divides trust into competence trust and goodwill trust when studying the behavior of subjects in electronic data trading systems.

In our study, trust includes three dimensions: competence, integrity, and goodwill. Competence trust refers to the knowledge and skills required by the trustee to perform a particular task (Colquitt et al., 2007). Integrity refers to the trustee's attributes, such as impartiality, fairness, consistency, and performance of commitments (Colquitt et al., 2007). Goodwill trust refers to the belief that the trustee will take mutual benefit practice as promised (Sako, 2006). Specifically, in the research on social responsibility, goodwill trust pertains to the degree of perceived altruism of corporate CSR initiatives (Joo et al., 2019). We expect that when perceived information quality gets higher, competence, integrity, and goodwill trust get higher.

Approval of a firm's ability to provide information increases consumer approval of the firm's goodwill (Nicolaou et al., 2013). The effort of firms to satisfy consumers' demand for information quality shows their sincerity toward consumers. This positive perception will inertially arouse the perception of corporate goodwill (Nooteboom, 2001). Therefore, the high level of information quality perception increases the goodwill perception of firms.

**Hypothesis 2a**. Perceived information quality will positively influence goodwill.

The more consumers feel that the information provided by firms is accurate and reliable, the more they believe in the professionalism and capabilities of enterprises. Consumer recognition of a firm's ability to provide information will increase trust in the firm's practical ability (McKnight et al., 2002). Therefore, we hypothesize that consumers will trust a firm's capabilities more when they perceive a high level of information quality.

**Hypothesis 2b**. Perceived information quality will positively influence competence.

When consumers perceive the enterprise's ability to provide information, they will believe its commitment to reaching a deal (Brownlie and Howson, 2005). Consumers can trust firms to provide high-quality products and services for a successful transaction. A high level of information quality is likely to translate into trust in the firm's products and services.

**Hypothesis 2c**. Perceived information quality will positively influence integrity.

### Trust beliefs and indirect reciprocity

Trust is often used to analyze human behavior, especially one party's perception of the other (Marková and Marková, 2004). According to the indirect reciprocity theory, trust belief is the antecedent factor of indirect reciprocity. Trust in the firm reduces the perception of uncertainty and maximizes the transmission of information to consumers, affecting their decisions (Colquitt et al., 2007).

We will analyze the relationship between the two based on the three dimensions of trust. Trust(competence, integrity, goodwill) can promote the relationship between the two parties (Dowell et al., 2015). Goodwill trust emphasizes trust in the altruistic tendencies of the trustee (Park and Tussyadiah, 2020). Trust in goodwill is based on the perception of the other party's sincere concern and intention to actively undertake social responsibilities (Lui and Ngo, 2004). It reduces the questioning of corporate care or reciprocal decisions (Das and Teng, 2001). Especially in the context that firms generally communicate with consumers by revealing information through commodity web pages, consumers' goodwill trust in the enterprise directly affects the purpose of communication. We propose the following hypothesis:

**Hypothesis 3a**. Goodwill trust will positively influence indirect reciprocity.

Competence trust is one of the keys to unlocking trust intention, which often activates subsequent effective or positive perception (Ibrahim and Ribbers, 2009). Twyman et al. (2008) point that competence trust can promote cooperative intentions in risk communication. Therefore, we expect that trust in firm-specific skills (e.g., the ability to implement [helping farmers program]) will increase consumers' positive emotions and attitudes toward the firm (e.g., indirect reciprocity).

**Hypothesis 3b**. Competence trust will positively influence indirect reciprocity.

In different types of transactions, integrity trust will have different effects from competence trust (Connelly et al., 2018). An increase in integrity trust implies a decrease in perceived opportunistic threats, whereas trust in competence does not (Lui and Ngo, 2004). Compared with the crisis of trust in competence, the crisis of integrity trust may lead to more severe consequences (Kim et al., 2004). Integrity is a virtue with utilitarian tendency (McKnight et al., 2002). Consumers' integrity perception of the firm will make them believe that it will provide high-quality services and products as promised and will not hide information or evade obligations (Saleh et al., 2013). Critcher and Gilovich (2010) point out that people gradually form attitudes based on perceptions. Consumers, perceiving the firm's integrity, can stimulate their choice of indirect reciprocity.

**Hypothesis 3c**. Integrity trust will positively influence indirect reciprocity.

### Perceived information quality and indirect reciprocity

The credibility of the information provided by the firm may influence the purpose of transparent information. Samu and Takács (2021) points out that credible information positively affects other people's decisions. Perceived information quality positively affects consumers' decision (Mannan et al., 2019). Trust is the mental mechanism through which influence arises (Cyr et al., 2010; Wang et al., 2016). Thus, we expect trusting beliefs to mediate the effects of PIQ on indirect reciprocity.

**Hypothesis 4a**. Goodwill trust will mediate the effects of perceived information quality on indirect reciprocity.
**Hypothesis 4b**. Competence trust will mediate the effects of perceived information quality on indirect reciprocity.
**Hypothesis 4c**. Integrity trust will mediate the effects of perceived information quality on indirect reciprocity.

### External factors associated with a trusted context

Although previous studies have shown the importance of compassion and the need for social status in understanding consumer prosocial behavior, there needs to be more exploration into other-oriented prosocial behavior driven by indirect reciprocity. Thus, their role in consumers' indirect reciprocity is still worth exploring.

#### The role of different compassion

Compassion has been a hot topic in psychology research for the past 30 years (Gilbert, 2017; Szcześniak et al., 2020). Human compassion is a cognitive process involving reflection on the past and predicting future behavior (Gilbert, 2019). Alternatively, it is the transformation of an emotional state into actual behavior. This emotion is triggered when people see others suffering through hardship and then choose to help those people (Goetz et al., 2010).

Compassion has individual differences. Compassionate people are more likely to feel the plight of others and be willing to help (Mayer et al., 1995). They pay more attention to the needs of others to establish a mutually supportive environment (Crocker and Canevello, 2008). They are also more willing to participate in and support projects that benefit social development (Horowitz et al., 2001; Crocker et al., 2009). Compassion affects how consumers view the responsible and irresponsible behaviors of enterprises (Xie et al., 2015).

Mayer et al. (1995) and Saslow et al. (2013) pointed out that compassion drives prosocial behaviors such as generosity. They view others with kindness, so they are more likely to trust others (Piferi and Lawler, 2006; Crocker and Canevello, 2008; Lemay Jr and Clark, 2008). An increase in compassion may predict an increase in trust (Colquitt et al., 2007; Crocker and Canevello, 2008). Liu and Wang (2010) and Nathoo et al. (2021) found that compassion promotes trust. Therefore, we propose the following possible hypotheses:

**Hypothesis 5a**. The effect of perceived information quality on goodwill trust is stronger for consumers with higher compassion.
**Hypothesis 5b**. The effect of perceived information quality on competence trust is stronger for consumers with higher compassion.
**Hypothesis 5c**. The effect of perceived information quality on integrity trust is stronger for consumers with higher compassion.

#### The role of different need for social status

Berger et al. (1980) and Anderson et al. (2015) argue that the need for social status is a basic human desire. The need for social status illuminates an individual's desire to increase their influence in a social group in order to gain the respect and admiration of others (Dubois et al., 2012). The need for social status emphasizes the psychological satisfaction that comes from the admiration and respect of others (Galinsky et al., 2008; Blader and Chen, 2011; Dubois et al., 2012; Kastanakis and Balabanis, 2012).

This psychological factor will influence the consumer's decision (Dreze and Nunes, 2009; Ordabayeva and Chandon, 2011; Correia et al., 2018, 2019). Zhang et al. (2018) indicated that facing CSR information, the high-level need for social status customers exhibits a more positive attitude than the low-level need for social status customers. Some consumers gain the respect of others by consuming products with prosocial attributes and sending prosocial signals to others (e.g., buying products with altruistic attributes Johnson et al., 2018).

Of course, they need to be convinced that the attributes of this product match their identity first (Ashforth and Mael, 1989). We seek consistency between our self-image and the image of the product we own. Just as consumers will use luxury goods to demonstrate their social status, we believe that consumers may also engage in so-called “conspicuous prosocial consumption” to show their alignment with certain prosocial beliefs (Johnson et al., 2018). Therefore, we hypothesize that consumers with high demand for moral sense (the need for social status) will be more willing to support the firm if they believe it is doing something prosocial.

**Hypothesis 6a**. The goodwill trust impact on indirect reciprocity is stronger for consumers with a higher need for social status.
**Hypothesis 6b**. The competence trust impact on indirect reciprocity is stronger for the consumer with a higher need for social status.
**Hypothesis 6c**. The integrity trust impact on indirect reciprocity is stronger for the consumer with a higher need for social status.

## Research methodology

### Construct measurement

Our design consists of a vignette experiment (based on three vignettes) and a post-experiment survey. To simulate an online shopping scenario, an online questionnaire will be used. The questionnaires will be distributed randomly to the subjects. This random assignment will make three scenarios appear in the face of a similar probability (Cook et al., 2002). This controls the homogeneity of other external factors in addition to the scene, which makes the results more credible. Random settings are such that there are no observable or unobservable differences other than the manipulated factor. Since treatment was the only aspect that differed between the groups, causal inferences were possible (Gerber and Green, 2012).

In the vignette experiment, participants were first told to imagine buying a box of walnut dates online. They would then be presented with a social commerce website for dates and walnut products. Each participant was provided with the same contextual information from the webpage banner: i.e., product images and names^1^. The above information is the same in all three scenarios; the only difference is the degree of transparency we want to manipulate. This ensures that our model is appropriately tested (Katok et al., 2018; Duan et al., 2021).

Such experiments are particularly appropriate when the dependent variable of interest is a perception (Miret et al., 2017; Lonati et al., 2018). We manipulate three levels of supply chain transparency to test the effects of supply chain transparency (see Table 1), which is captured by a categorical variable (0 = low supply chain transparency, 1 = medium supply chain transparency, 2 = high supply chain transparency). Transparency design is based on the practice in social commerce sites and the social responsibility report on the enterprise webpage. Then, we used items of indirect reciprocity to measure the effects of supply chain transparency on the consumer.

**Table 1:** The instructions of manipulation.

**Manipulation level**	**Content**
Low supply chain transparency	This product has the properties to help farmers.
Medium supply chain transparency	This product has the properties to help farmers. Gather love into the light. Light up, hope! We believe that everyone working in our extended supply chain should earn enough to maintain a decent standard of living. We hope that more unsalable agricultural products can be sold through the “Internet + brand” to do our part to improve farmers' income and brighten the future for more children.
High supply chain transparency	0.3% of each transaction will be fed back to the upstream docking farmers, and It has accumulated 37,473 transactions.
	This product has the properties to help farmers. Gather love into the light. Light up, hope! We believe that everyone working in our extended supply chain should earn enough to maintain a decent standard of living. We hope that more unsalable agricultural products can be sold through the “Internet + brand” to do our part to improve farmers' income and brighten the future for more children.
	To take effective and scientific support measures has, an investment in the native region covers an area of 300 acres of freeze-dried fruit and vegetable products processing base, with the “company + production base+ peasant household” mode, docking to the home to the poor people, be included in the company to the industry chain, the co-construction, and sharing system, both practical and improve farmers income. Details can be found on the company's official website and in the corporate social responsibility report and annual report disclosed on the Shenzhen Stock Exchange.

After reading the transparency manipulation script, the participants checked whether the transparency manipulation was successful through the response scale. Transparency manipulation test, using Höddinghaus et al. (2021)'s transparency scale to ask subjects, “I think I could understand the decision-making processes of [helping farmers program] very well through the business's decision-making process about [helping farmers program]. I think the decision-making processes of [helping farmers program] are clear and transparent” (1 = strongly disagree, 7 = strongly agree).

The questionnaire for the vignette experiment includes: brief instructions on the survey study; Collecting the background and demographic information of the participants (including age, gender, and education); Collecting experimental data for other perceptive variables (i.e., perceived information quality, competence, goodwill, and integrity). A seven-point Likert-type scale ranging from 1 (strongly disagree) to 7 (strongly agree) was constructed to measure the respondent agreement level on all the items. All scale items and the operationalization of the related constructs are summarized in Table 2.

**Table 2:** Measurement.

**Constructs**	**Items**	**References**
PIQ	The webpage provides sufficient information.	Yang, 2021
	The webpage provides reliable information.	
	The webpage provides precise information.	
	The information contained in the product webpage meets my needs.	
IR	I am grateful for what businesses are doing for farmers.	Szcześniak et al., 2020
	I appreciate how companies help vulnerable people in the supply chain.	
	I would love to do something for enterprises that adopt farming practices.	
TC	I believe that the merchant is efficient in their support for [helping farmers program].	McKnight et al., 2002,
	I believe that the merchant is experienced in their support for [helping farmers program]	Gharib et al., 2019
	I believe that the merchant is professionally in their support for [helping farmers program]	
TI	I trust the service level of the merchant.	McKnight et al., 2002,
	I believe in the quality of the product.	Gharib et al., 2019
	I believe in the integrity of the merchant.	
TG	The merchant is hard work and philanthropic in their support for [help farmers program].	Joo et al., 2019
	Merchants treat farmers fairly when purchasing their produce because they care [helping farmers program].	
	The merchant is acting benevolently in their support for [helping farmers program].	
NS	I want my peers to respect me and hold me in high esteem.	Park et al., 2017,
	Being a highly valued member of my social group is important to me.	Flynn et al., 2006
	I would like to cultivate the admiration of my peers.	
C	If I see someone going through a difficult time, I try to be caring toward that person.	Pommier et al., 2020
	I notice when people are upset, even if they don't say anything.	
	Everyone feels down sometimes, and it is part of being human.	
	I tend to listen patiently when people tell me their problems.	
	When others feel sadness, I try to comfort them.	

PIQ, Perceived information quality; NS, Need for social status; C, Compassion; IR, Indirect reciprocity; TC, Competence trust; TI, Integrity trust; TG, Goodwill trust.

In the post-experiment survey, we set some questions to check whether the subjects read and filled out the questionnaires carefully. Depending on the choice of subjects, we will identify and eliminate some invalid questionnaires. Questions mostly ask for illustrated information or simple statements, for which there is a fixed answer.

Although our research is not cross-national, we still refer to the methods of Collaborative and Iterative Translation (Douglas and Craig, 2007) and Back translation (Behr, 2017) regarding the translation of the questionnaire. First, two bilingual speakers whose native language is Chinese translated the English scale into Chinese. Then, it was sent to two experts in that field for review. Afterward, 30 consumers were randomly recruited online and asked about their opinions on each questionnaire item. Comprehension, to see if there is any deviation in semantic understanding; finally, it was translated into English by two bilingual staff whose native language is English. The final English-language questionnaire was then compared with the original version to make the scale easier for respondents to understand without deviating from the original meaning of the items. Certain steps in this process are iterated to ensure translation equivalence (Teo and Liu, 2007).

### Data collection

In order to ensure that real people fill in the questionnaire, we choose to recruit WeChat users as subjects. There is a high degree of convergence between groups using WeChat and those shopping on social networking platforms. Due to the higher frequency of daily use of WeChat, a higher questionnaire response rate can also be provided. WeChat is China's largest social media platform, with over 1 billion monthly active users in 2020 (Dragon Social Reporting)^2^. “The average user still accesses Moments over ten times daily, making for roughly 10 billion hits every 24 h” (TechNode Briefing)^3^. Shao and Pan (2019) and Guo et al. (2020) also recruited respondents through WeChat.

The questionnaire was collected in July 2020. In the formal survey, 860 people were recruited to participate, and 607 valid questionnaires were collected (0.71). The respondent characteristics are depicted in Table 3.

**Table 3:** Respondent characteristics (*N* = 607).

**Demographic**		**Frequency**	**Percent**
Age	18–25	308	0.51
	26–35	249	0.41
	36–45	50	0.08
Gender	Male	328	0.54
	Female	279	0.46
Education	High school	81	0.13
	Junior college	101	0.17
	Undergraduate	274	0.45
	Postgraduate or higher	151	0.25

The variables' difference significance between the first 30% and the final 30% responses (Wu et al., 2022) evaluated the nonresponse bias. The Paired sample t-Test was not significant (*p*>0.05) in Table 4. The nonresponse bias does not affect the conclusions of our study.

**Table 4:** Analysis of non-response bias.

	**The first 30%**	**The final 30%**	**Significance**
PIQ	4.525	4.460	0.874
TG	4.696	4.555	0.385
TC	4.692	4.595	0.204
TI	4.661	4.643	0.621
IR	5.037	5.044	0.943
C	5.553	5.567	0.863
NS	5.178	5.154	0.810

The first 30% refers the first 30% responses; The final 30% is the final 30% responses. PIQ, Perceived information quality; NS, Need for social status; C, Compassion; IR, Indirect reciprocity; TC, Competence trust; TI, Integrity trust; TG, Goodwill trust.

Before the model analysis, we examined the manipulation effect of supply chain transparency as mentioned in the method. We evaluate the efficacy of the supply chain transparency manipulation by one-way ANOVA. The results show that the Welch test is significant (*F* = 38.050, *p* < 0.001). The mean difference is significant at the 0.05 level by *post-hoc* tests (Tamhane's T2 Test in Table 5). Thus, the manipulation of supply chain transparency has the main effect on the supply chain transparency response.

**Table 5:** Multiple comparisons.

**Dependent variable: Supply chain transparency**	**(I)Trans**	**(J)Trans**	**MD (I-J)**	**Std. Error**	***P*-value**
Tamhane's T2	0(*n* = 206)	1	–10.142	2.434	0.000
		2	–19.297	2.220	0.000
	1(*n* = 194)	0	10.142	2.434	0.000
		2	–9.155	2.572	0.001
	2(*n* = 207)	0	19.297	2.220	0.000
		1	9.155	2.572	0.001

MD, Mean difference.

### Statistical analysis

We used structural equation modeling (SEM) based on SmartPLS 3.0 to analyze the measurement and path model. Although indirect reciprocity theory is often used for explaining altruistic behavior, indirect reciprocity has never been studied in the consumer behavior field. PLS does not require the data to conform to a normal distribution, which is more friendly to models with multiple variables (Goodhue et al., 2012). It is also often used to analyze newly developed models or to conduct exploratory analysis (Henseler, 2018). Therefore, the PLS approach was more suited to our study.

## Data analysis

### Quality of measurement model

We assessed the reliability, convergent validity, and discriminant validity of the latent variables in the measurement model. Table 6 shows the factor loadings, composite reliability, Cronbach's alpha, and average variance extracted (AVE) of all of the constructs in the model. Table 7 illustrates the correlation analysis of latent variables and the square root of the AVE.

**Table 6:** Measurement quality model.

**Constructs**	**Items**	**Factor** **Loading**	**Cronbach's** **alpha**	**Composite reliability**	**Average** **variance** **extracted (AVE)**
Perceived information quality (PIQ)	PIQ1	0.89	0.92	0.94	0.80
	PIQ2	0.88			
	PIQ3	0.91			
	PIQ4	0.90			
Goodwill trust (TG)	TG1	0.88	0.86	0.92	0.78
	TG2	0.88			
	TG3	0.90			
Competence trust (TC)	TC1	0.92	0.90	0.94	0.83
	TC2	0.90			
	TC3	0.92			
Integrity trust (TI)	TI1	0.91	0.90	0.94	0.83
	TI2	0.90			
	TI3	0.93			
Indirect reciprocity (IR)	IR1	0.85	0.73	0.85	0.65
	IR2	0.86			
	IR3	0.70			
Need for social status (NS)	NS1	0.82	0.74	0.85	0.65
	NS2	0.81			
	NS3	0.80			
Compassion (C)	C1	0.82	0.86	0.90	0.65
	C2	0.81			
	C3	0.78			
	C4	0.81			
	C5	0.80			

**Table 7:** Correlation analysis of latent variables and square root of the AVE.

	**C**	**IR**	**PIQ**	**SD**	**TG**	**TC**	**TI**
C	**0.80**						
IR	0.50	**0.81**					
PIQ	0.29	0.71	**0.89**				
NS	0.48	0.54	0.47	**0.81**			
TG	0.39	0.73	0.84	0.45	**0.89**		
TC	0.39	0.69	0.80	0.46	0.87	**0.91**	
TI	0.45	0.67	0.77	0.44	0.85	0.81	**0.91**

NS, Need for social status; C, Compassion; IR, Indirect reciprocity; TC, Competence trust; TI, Integrity trust; TG, Goodwill trust. The bold values indicate the square root of the AVE.

For each latent variable, the item falls within the acceptable range. The factor loadings for all indicators were larger than the recommended threshold of 0.7 (Hair et al., 2019). The composite reliability (CR) and Cronbach's alpha were examined to assess the items' level of reliability. Reliability reflects the consistency between items that measure the same construct (Weir, 2005). Both Cronbach's alpha and composite reliability are >0.7. Then the reliability coefficients are acceptable (Cronbach, 1951; Hair et al., 2010).

Table 6 shows the composite reliability score ≥0.85, and the Cronbach's alpha value ≥0.73, indicating acceptable internal reliability. Convergent validity measures the correlation between measurement scales and construct and is assessed by the AVE of each construct. The AVEs are all above 0.50, indicatingng a reasonable convergent validity of the constructs (Pavlou and Fygenson, 2006; MacKenzie et al., 2011; Hair et al., 2019; 2021).

In Table 7, the diagonal elements are the square root of the AVEs, all of which were found to be greater than the correlation coefficients with other constructs. Discriminant validity was thus verified.

The cross loading criterion meets subjective independence.Cross Loading Criterion-subjective independence can help reduce the presence of multicollinearity amongst the latent variables. The average variance extracted (AVE) of a latent variable is higher than the squared correlations between the latent variable and all other variables (Chin, 2010), (see Table 8). Specifically, the differences between loadings on principle factors and other constructs are all higher than the threshold (i.e., 0.1) (Gefen and Straub, 2005).

**Table 8:** Factor loadings and cross-loadings.

	**Compassion**	**Indirect reciprocity**	**Perceived information quality**	**Need for social status**	**Goodwill trust**	**Competence trust**	**Integrity trust**
C1	**0.818**	0.419	0.29	0.39	0.352	0.33	0.401
C2	**0.813**	0.447	0.28	0.37	0.385	0.418	0.406
C3	**0.783**	0.353	0.153	0.392	0.233	0.225	0.296
C4	**0.806**	0.382	0.176	0.372	0.258	0.263	0.312
C5	**0.799**	0.393	0.224	0.394	0.278	0.291	0.357
IR1	0.401	**0.852**	0.572	0.423	0.58	0.556	0.547
IR2	0.372	**0.864**	0.698	0.413	0.708	0.664	0.63
IR3	0.466	**0.701**	0.409	0.48	0.454	0.439	0.429
PIQ1	0.26	0.66	**0.889**	0.402	0.735	0.698	0.685
PIQ2	0.302	0.632	**0.884**	0.425	0.75	0.711	0.709
PIQ3	0.232	0.614	**0.907**	0.434	0.782	0.748	0.702
PIQ4	0.242	0.618	**0.895**	0.433	0.728	0.72	0.666
NS1	0.384	0.42	0.376	**0.823**	0.338	0.348	0.351
NS2	0.467	0.492	0.365	**0.808**	0.362	0.392	0.35
NS3	0.277	0.369	0.417	**0.796**	0.404	0.383	0.366
TG1	0.369	0.692	0.718	0.411	**0.881**	0.769	0.729
TG2	0.291	0.621	0.778	0.397	**0.878**	0.747	0.712
TG3	0.365	0.62	0.73	0.392	**0.897**	0.794	0.812
TC1	0.345	0.624	0.749	0.414	0.808	**0.916**	0.743
TC2	0.376	0.655	0.727	0.428	0.789	**0.903**	0.742
TC3	0.355	0.621	0.73	0.426	0.786	**0.922**	0.735
TI1	0.421	0.635	0.716	0.462	0.786	0.75	**0.908**
TI2	0.386	0.577	0.703	0.353	0.764	0.734	**0.902**
TI3	0.422	0.62	0.696	0.382	0.769	0.733	**0.927**

PIQ, Perceived information quality; NS, Need for social status; C, Compassion; IR, Indirect reciprocity; TC, Competence trust; TI, Integrity trust; TG, Goodwill trust. The bold values indicate the factor loadings.

The results suggest that the hypothesized constructs have no major measurement issues.

### Common method variance bias

Since our research data were all self-reported and were a single data source, common method bias could be present. Common method bias can be categorized into two types: ex ante(questionnaire design) and ex post (statistical controls) (MacKenzie and Podsakoff, 2012; Viswanathan and Kayande, 2012). For ex-ante, our questionnaire design abides by the principles (Giving clear instructions, ensuring anonymity of responses, keeping the survey short, and minimizing redundant measures and overlap) (Podsakoff, 2003; Baumgartner and Weijters, 2012; Viswanathan and Kayande, 2012). In addition, we adopt the proximal separation (Garg, 2019). Finally, We add a marker variable to the survey to use the marker technique in ex-post (Lindell and Whitney, 2001). In ex-post, we conduct Harman's single-factor test for common method bias (Ylitalo, 2009).

In our results, more than one factor emerged, and the highest level of covariance explained by one factor was < 50% (Fuller et al., 2016). This means that no single latent variable could account for all indicators. Nevertheless, Some researchers (e.g., Podsakoff and Organ, 1986; Lindell and Whitney, 2001; Podsakoff, 2003; Podsakoff et al., 2012) pointed out that Harman's test to assess common method bias was insufficiently sensitive. Thus, to solve the potential CMV issue in our studies, we followed the CFA marker variable approach and the unmeasured standard method variable approach (Podsakoff, 2003; Williams et al., 2010) to test the influence of CMV, which are widely used in the literature (e.g., Wu et al., 2010; Li et al., 2022). We found that CMV did not pose a threat to our model. Detailed results are provided upon request.

### Structural path analysis

**Figure 3:**
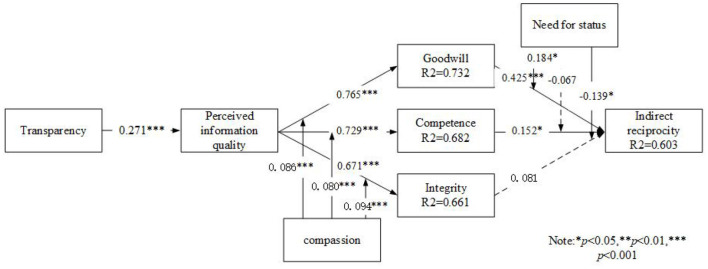
SmartPLS analysis results of the research model. ^*^: *p* < 0.05; ^**^: *p* < 0.01; ^***^: *p* < 0.001.

We selected PLS to test our research model. SmartPLS 3.0 was used for analyzing the path relationship between constructs. We use the bootstrap resampling method (using 5,000 random samples from the data points of the collected data set) to estimate the path significance coefficient levels (Wu et al., 2022). Figure 3 illustrates the results of the structural model analysis. The final model explains a substantial portion of the variance, with a coefficient of determination (*R*^2^) of 0.732 for goodwill, 0.682 for competence, 0.661 for integrity, and 0.603 for indirect reciprocity as a dependent variable, indicating that the research model has significant explanatory power (Hulland, 1999; Gefen et al., 2000).

Transparency has a significant direct effect on perceived information quality (β = 0.271; *p* < 0.001), supporting H1. Perceived information quality can be seen to have a positive impact on goodwill (β = 0.765; *p* < 0.001), competence (β = 0.729; *p* < 0.001) and integrity (β = 0.671; *p* < 0.001), in support of H2a, H2b, and H2c. As hypothesized, there is a relationship between trust and indirect reciprocity. Specifically, goodwill (β = 0.425; *p* < 0.001), competence (β = 0.152; *p* < 0.05) and integrity (β = 0.081; *p*>0.05) are shown to lead to indirect reciprocity, supporting H3a and H3b, but not H3c.

#### Mediation effect test

This study adopts the method described by Nitzl et al. (2016) to test whether trust belief (goodwill, competence, and integrity) fully or partially mediates the relationship between perceived information quality and indirect reciprocity. Many scholars followed a procedure similar to that proposed by Baron and Kenny (1986) for multiple regression analysis in PLS. However, scholars have recently questioned the accuracy of Baron and Kenny (1986)'s mediation test method (Shrout and Bolger, 2002; Preacher and Hayes, 2004, 2008; Zhao et al., 2010). Because PLS can test mediating effects in a single model at once, a step-wise approach is not necessary (Nitzl et al., 2016).

Therefore, we follow Wongkitrungrueng and Assarut (2020) and use a bootstrap method to test the mediation effect of trust belief. Finally, we performed the mediation analysis based on 5,000 bootstrapped samples and computed bias-corrected 95% confidence intervals (CIs). Results of indirect/mediating effects are summarized in Table 9.

**Table 9:** Mediation effect test.

**Path**	**Indirect effect**	**Bootstrapping** **Bias-corrected 95** **% CI**		**Results**
		**Lower**	**Upper**	
PIQ → TG → IR	0.326^***^	0.227	0.424	H4a is supported
PIQ → TC → IR	0.111^*^	0.022	0.201	H4b is supported
PIQ → TI → IR	0.055	-0.019	0.132	H4c is not supported

PIQ, Perceived information quality; IR, Indirect reciprocity; TC, Competence trust; TI, Integrity trust; TG, Goodwill trust. ^*^: *p* < 0.05; ^**^: *p* < 0.01; ^***^: *p* < 0.001.

As shown in Table 9, for the link PIQ → TG → IR, the indirect path is significant (95 % CI [0.227, 0.424]), as the CI interval does not contain zero, indicating that Goodwill trust mediates the relationship between perceived information quality and indirect reciprocity. Thus, H4a is supported. Similarly, for the PIQ → TC → IR link, the indirect path is also significant (95 % CI [0.022, 0.201]), indicating that competence also partially mediates the relationship between perceived information quality and indirect reciprocity.

Therefore, H4b is supported. However, for the link PIQ → TI → IR, the indirect path is nonsignificant (95 % CI [-0.019, 0.132]), indicating that integrity does not mediate the relationship between perceived information quality and indirect reciprocity. Therefore, H4c is not supported.

#### Moderation effect test

This section provides the results of statistically testing moderating effects based on the need for social status and compassion in our research model. Table 10 offers more details of testing moderating effects using complete bootstrapping in SmartPLS. The results demonstrate: the positive moderating effect of compassion on the relationship between perceived information quality and goodwill (β = 0.086; *p* < 0.001), competence (β = 0.080; *p* < 0.001) and integrity (β = 0.094; *p* < 0.001) were significant, providing evidence in support of H5a, H5b, and H5c.

**Table 10:** Bootstrap moderation analysis: Need for social status and compassion.

					***CIBC***	
	**β**	***SM***	***SD***	***p***	**2.50%**	**97.50%**
NS*TG → IR	0.184	0.180	0.073	0.0115	0.040	0.323
NS*TC → IR	-0.067	-0.067	0.067	0.3175	-0.203	0.063
NS*TI → IR	-0.139	-0.135	0.059	0.0194	-0.255	-0.023
C*PIQ → TG	0.086	0.086	0.021	0.0000	0.046	0.126
C*PIQ → TC	0.080	0.079	0.023	0.0006	0.035	0.126
C*PIQ → TI	0.094	0.093	0.028	0.0009	0.038	0.150

*SM* is Sample Mean; *SD* is Standard Deviation; *p* is *p*-value; *CIBC* is confidence intervals bias corrected. PIQ, Perceived information quality; NS, Need for social status; C:,Compassion; IR, Indirect reciprocity; TC, Competence trust; TI, Integrity trust; TG, Goodwill trust.

Moreover, we also found that the positive moderating effect of the need for social status on the relationship between goodwill and indirect reciprocity was significant (β = 0.184; *p* < 0.05), providing evidence in supporting H6a. However, the negative moderating effect of the need for social status on the relationship between competence (β = −0.067; *p*>0.05) and indirect reciprocity was nonsignificant. H6b is not supported. Then, we also found that the negative moderating effect of the need for social status on the relationship between integrity (β = −0.139; *p* < 0.05) and indirect reciprocity was nonsignificant, revealing the opposite effect H6c.

**Figure 4:**
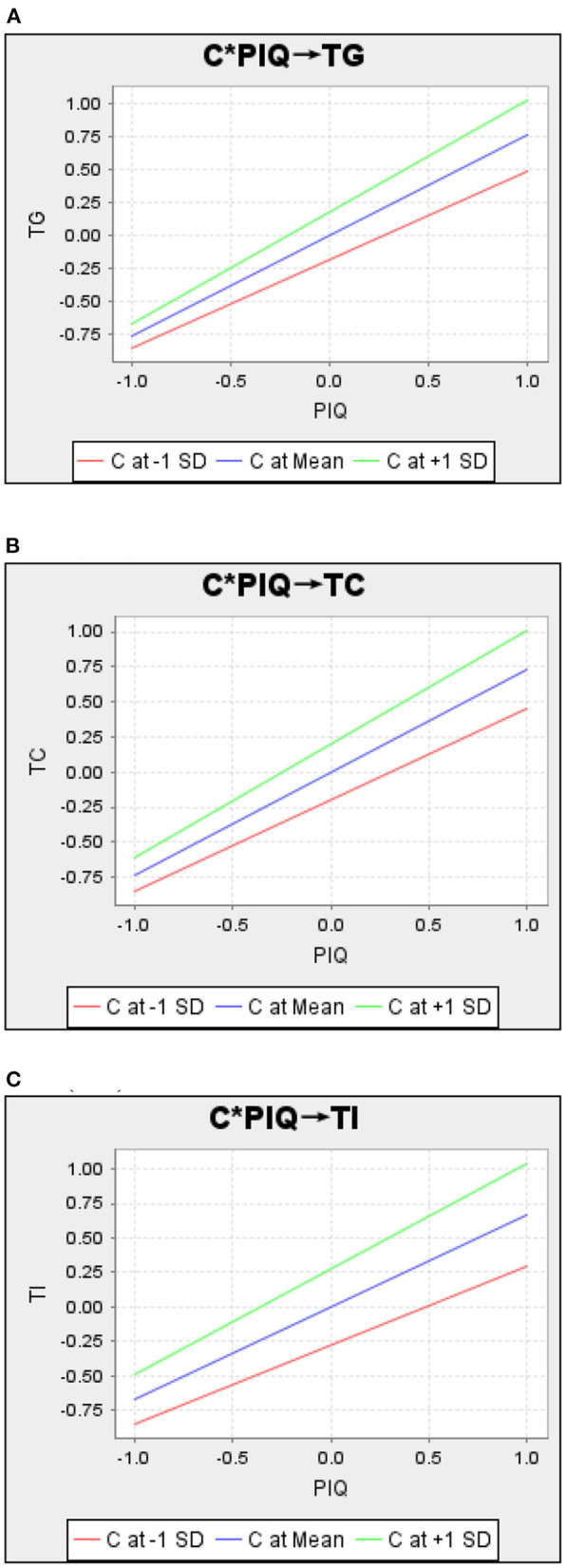
Interaction of perceived information quality and compassion. **(A)** Compassion (C) moderates the relationship between perceived information quality (PIQ) and Goodwill trust (TG). **(B)** Compassion (C) moderates the relationship between perceived information quality (PIQ) and competence trus (TC). **(C)** Compassion (C) moderates the relationship between perceived information quality (PIQ) and integrity trust (TI).

**Figure 5:**
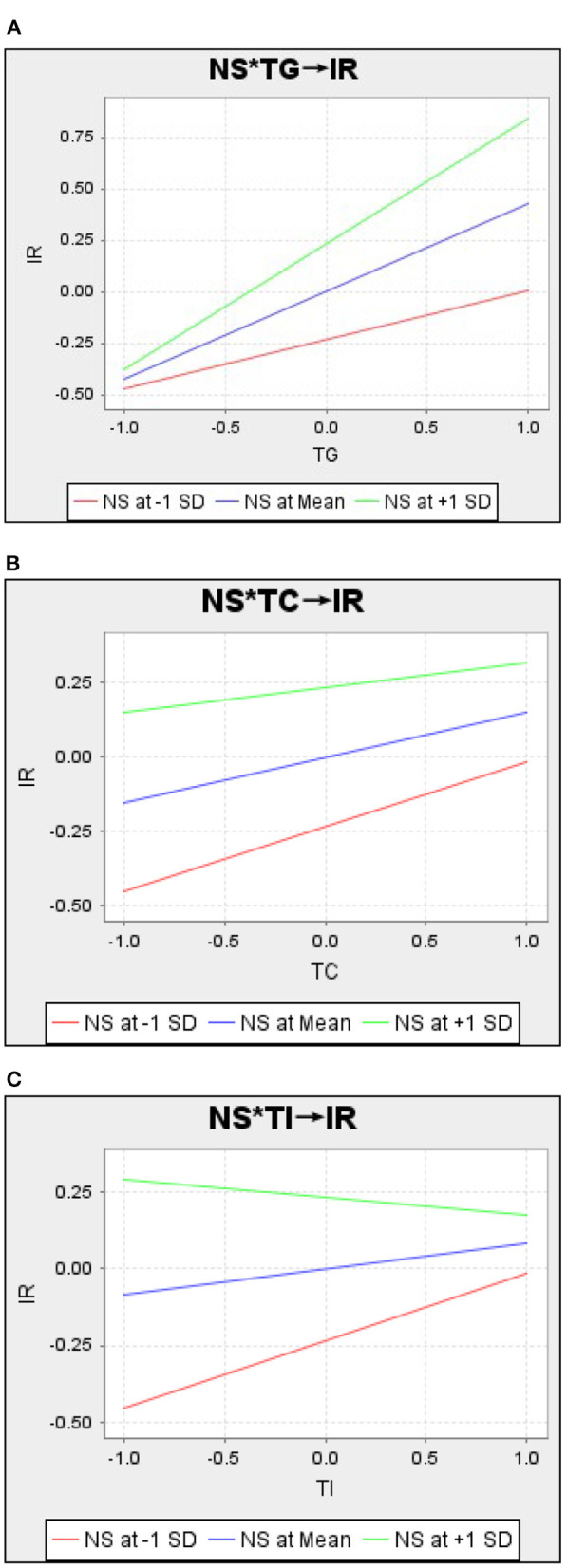
Interaction of trust and need for social status. **(A)** Need for social status (NS) moderates the relationship between goodwill trust (TG) and indiect reciprocity (IR). **(B)** Need for social status (NS) moderates the relationship between competence trust (TC) and indiect reciprocity (IR). **(C)** Need for social status (NS) moderates the relationship between integrity trust (TI) and indiect reciprocity (IR).

The moderating effect of compassion and the need for social status is further examined in Figures 4, 5. According to Figure 4, the slopes of the three lines are as follows: the green line (NS at +1 SD) > the blue line (NS at Mean) > and the red line (NS at –1 SD), demonstrating that compassion positively moderates the relationship between perceived information quality and goodwill, competence, and integrity. That is, as perceived information quality rises, consumers with a high level of compassion are more likely to display a high level of goodwill, competence, and integrity trust than those with a lower level of compassion.

According to Figure 5 (NS*TG->IR), the slopes of the three lines are as follows: the green line (NS at +1 SD) > the blue line (NS at Mean) > and the red line (NS at –1 SD), demonstrating that at high, compared to low, levels of goodwill trust, consumers high in the need for social status, compared to those low in the need for social status, are more likely to exhibit indirect reciprocity. In Figure 5 (NS*TC->IR), the slopes of the three lines are as follows: the green line (NS at +1 SD) < the blue line (NS at Mean) < and the red line (NS at –1 SD), demonstrating that consumers with a low need for social status are more likely to exhibit indirect reciprocity in the case of high competence trust than in the case of low competence trust, compared to consumers with a high need for social status.

Then, in Figure 5 (NS*TI->IR), we find an interesting phenomenon: the slopes of the three lines are as follows: the green line (NS at +1 SD) < 0, and the blue line (NS at Mean) < the red line (NS at –1 SD). The effect of integrity on indirect reciprocity is reversed for consumers with a low need for social status and for consumers with a high need for social status relative to the general group. Consumers with a lower need for social status, as opposed to the general group, are more likely to exhibit indirect reciprocity in the case of higher integrity trust than in the case of lower integrity trust. Consumers with a higher need for social status, as opposed to the general public, are more likely to exhibit indirect reciprocity in the case of lower integrity trust than in the case of higher integrity trust.

## Discussion

In the context of digital agriculture and social commerce, we expect to examine the theoretical mechanism of “Social Commerce Helping Farmers” in China's poverty alleviation project from a market perspective. We found that Chinese consumers have positive attitudes toward companies participating in “Social Commerce Helping Farmers” projects and are willing to reward companies for participating in such socially responsible behavior.

The higher the transparency of the supply chain provided by the enterprise, the higher the quality of information consumers perceive, and the more willing they are to trust the enterprise. The empirical results show that supply chain transparency significantly affects perceived information quality, and perceived information quality significantly impacts consumer trust in three dimensions. The supply chain transparency of corporate social responsibility programs will have a stronger impact on consumers' trust in the company's goodwill and ability to implement programs. We find that perceived information quality is different extent effect to three dimensions of trust beliefs [goodwill (β = 0.765; *p* < 0.001)>competence (β = 0.729; *p* < 0.001) >integrity (β = 0.671; *p* < 0.001)].

The difference also exists between consumer trust and perceptions of indirect reciprocity. Consumers' trust in the goodwill of companies and in their ability to implement “Social Commerce Helping Farmers” can translate into indirect reciprocity for companies. However, trust in the quality of services and products enterprises provide cannot be translated into indirect reciprocity. Goodwill (β = 0.425; *p* < 0.001), competence (β = 0.152; *p* < 0.05) and integrity (β = 0.081; *p*>0.05) differently affect indirect reciprocity.

Consumer heterogeneity also asymmetrically affects the role of the three dimensions of trust. For consumers with high compassion, when they feel that the information provided by the company is of high quality, they will convert the recognition of the information into more trust in the service and quality of the company (integrity trust).

We found an interesting phenomenon regarding the moderating effect of the need for social status. Consumers with different levels of need for social status exhibit different levels of indirect reciprocity in the face of different dimensions of trust. When faced with higher levels of goodwill trust, consumers with a higher need for social status demonstrate higher levels of indirect reciprocity than consumers with a lower need for social status. When faced with higher levels of competence trust, consumers with a lower need for social status demonstrate higher levels of indirect reciprocity than consumers with a higher need for social status. Higher integrity trust exhibits greater indirect reciprocity for consumers with a lower-than-average level of need for social status. However, for consumers with above-average social status needs, higher integrity trust exhibits lower indirect reciprocity.

In view of this, we propose that the contradictions found in previous studies regarding the effects of the need for social status on trust and pro-social behavior can be attributed to the different dimensions of the variables involved. At the same time, we presume that consumers with high social status needs will be more sensitive and have more pro-social tendencies under a pure altruistic dimension. However, when this altruistic tendency is weaker, consumers with a high need for social status will have a higher decrease in pro-social tendencies than consumers with a low need for social status. Thus, in the case of higher competence trust, consumers with a low need for social status will exhibit higher indirect reciprocity than those with a high need for social status. Also, in integrity trust, the same is true to the extent that consumers with a high need for social status exhibit resistance to indirect reciprocity.

### Theoretical contributions

Our research provides a theoretical mechanism for analyzing the “Social Commerce Helping Farmer” project from a market perspective. According to the process of indirect reciprocity, we construct a research model of China's “Social Commerce Helping Farmer” project and propose a new framework for analyzing consumers' prosocial behavior. This, in turn, enriches research on supply chain transparency, perceived information quality, consumer trust, indirect reciprocity, and consumer heterogeneity (compassion and need for social status).

Our research complements the transparency of socially responsible supply chains in the context of China's “Social Commerce Helping Farmer.” Different from the previous transparency focus on uniqueness of product quality (Sammer and Wüstenhagen, 2006; Saberi et al., 2019), cost (Bai and Sarkis, 2020), nature of labor conditions in supplier factories (Leitch, 2017) and sustainable production (Garcia-Torres et al., 2019). Results support supply chain transparency as a pre-variable for perceived information quality.

The successful identification of perceived information quality antecedents enriched the existing theories on perceived information quality. Previous studies verified the significant impact of information disclosure and type on consumers' information perception (Gefen and Straub, 2000; Zhou et al., 2018). This study further analyzes how the CSR practice transparency affects consumers' perceived information quality.

Our study not only enriched the existing empirical research on perceived information quality but also further proved the value of perceived information quality in pre-conversion into consumer trust (goodwill trust, competence trust, integrity trust) (Nicolaou and McKnight, 2006; Sarkar et al., 2020; Talwar et al., 2020). Although perceived information quality is considered a reliable perspective for predicting consumer trust, few studies have separately explored the correlation of perceived information quality with trust's three dimensions (goodwill, competence, and integrity). This is consistent with previous studies about different types of trust in networks (Silic and Ruf, 2018; Talwar et al., 2020).

It not only supplements the practical application of indirect reciprocity theory but also takes indirect reciprocity variables as research objects for the first time. According to the indirect reciprocity formation process, supply chain transparency serves as a clue and stimulus source; information quality and trust are psychological perceptions; and consumers' indirect reciprocity intention is the result of consumers' indirect reciprocity. The indirect reciprocity theory has been studied for decades, but it remains almost exclusively a theoretical topic (Nowak and Sigmund, 2005). Our study fills a gap in empirical research on indirect reciprocity in the context of consumer motivation.

Based on the research framework of indirect reciprocity theory, we explore the boundary conditions of the model. This will complement the study of the influence of consumer compassion and the need for social status on consumer decision processes. It explicitly complements research on how compassion moderates the relationship between perceived information quality and trust and how the need for social status moderates the relationship between trust and indirect reciprocity.

We hypothesize that the interaction between compassion and perceived information quality asymmetrically affects the three dimensions of consumer trust. That is, compassion divides consumers into different groups, and perceived information quality is the factor that motivates trust. We explain the interaction between the need for social status and trust by treating the need for social status as a trigger for differences in trust across dimensions and trust as a stimulus for indirect reciprocity.

According to context, provide a theoretical basis for China's “Social Commerce Helping Farmers” project and participating enterprises. If we want to achieve the goal of alleviating poverty and not returning to poverty, we need to give full play to the incentive role of the market. Consumers' positive response to companies with high transparency in the “Social Commerce Helping Farmers” project will promote the enthusiasm of enterprises to help farmers and the transparent “Social Commerce Helping Farmers” project. The positive interaction between the two makes the social responsibility practice of helping farmers form a virtuous cycle and sustainable development. This study has positive practical implications in the current context of concerns about farmers returning to poverty, the persistence of farmers seeking help, and a general lack of consumer trust.

### Implications for practice

We suggest that managers attempting to use social media communication to foster consumer trust must pay attention to supply chain transparency and consumer-perceived information quality. To cultivate consumer trust, managers should actively participate in corporate social responsibility activities, improve supply chain transparency, and give consumers a positive perception of information quality. Operators should also encourage transparency in socially responsible practices, create an atmosphere of trust between consumers and businesses, and inspire consumers to pay attention to and think deeply about businesses.

In addition, managers should recognize that in social commerce, the communication of different types of supply chain information by merchants can affect consumers' trust in goodwill, competence, and integrity differently. Focusing on communicating more social responsibility messages to companies with a reputation for excellent service and quality will increase consumer trust.

Consumers' indirect reciprocity decisions are more sensitive to goodwill trust. This means that if managers want to reduce consumers' sensitivity to commodity prices (increase indirect reciprocity), they must pay attention to the disclosure of information on altruistic behaviors such as corporate social responsibility practices. Be adept at using web technology to provide authoritative methods of information verification and communicate with consumers at all times about what they expect from the supply chain.

Third, by displaying ethical behavior in the supply chain on social commerce platforms, companies can not only activate customers' pro-social consciousness, increase positive consumer response to companies, and guide consumers to purchase pro-social goods but also attract consumers with pro-social values. In addition, companies can remind consumers in their messages that purchasing pro-social goods brings external social rewards and internal emotional satisfaction (e.g., the need for social status and compassion).

Finally, our study may provide practical guidance for sustainable entrepreneurship. The social responsibility initiative of helping farmers belongs to environmental, social, economic, and fair issues in corporate sustainability (Amini and Bienstock, 2014). Our articles may contribute to research related to sustainable entrepreneurship. The core of its business model is sustainability orientation (Schaltegger et al., 2016), which can capture economic value while solving economic, ecological, and societal problems (Schaltegger and Wagner, 2011). Our research highlights the role of corporations (Patzelt and Shepherd, 2011) in addressing the non-economic benefits of social development. At the same time, in the context of specific industry projects, we empirically analyze the relation mentioned by Centobelli et al. (2022) between technological capabilities (based on web 4. 0 supply chain transparency) and supply chain practices.

### Limitations and future research directions

The sample group we recruited comprises young and middle-aged people with higher education levels, which is a relatively limited sample. Although the respondents were users with some experience using social commerce, future research may invite subjects from a more diverse population to increase the generalizability of the findings.

We examine the predictive effect of consumer trust on indirect reciprocity in “Social Commerce Helping Farmers.” In other situations or for other research subjects, future research could consider the impact of other perceptual factors on indirect consumer reciprocity and additional boundary conditions.

While comprehensive, this study is only an initial foray into the field. Future researchers should conduct in-depth research from multiple perspectives. For example, this study only discusses consumers' indirect reciprocity pre-variable. This study focuses mainly on the process by which consumers generate indirect reciprocal intentions without extending to the actual indirect reciprocal behavior. In other words, our research is still stuck in the measurement of intention. Future research may analyze consumers' indirect reciprocity behavior in actual purchase situations to measure the impact of firms making prosocial practices transparent to consumers.

## Data availability statement

The raw data supporting the conclusions of this article will be made available by the authors, without undue reservation.

## Ethics statement

The studies involving human participants were reviewed and approved by Academic Committee of School of Economics and Management, South China Agricultural University. Written informed consent for participation was not required for this study in accordance with the national legislation and the institutional requirements.

## Author contributions

SH writing–original draft, reviewing, editing, and contributed to the article and approved the submitted version.

## Footnotes

^1^In order to get as close as possible to the actual scenario of merchants selling agricultural products and identify the more realistic decision-making process of the subjects. We simulated and designed our experimental scenario by sorting out the patterns of merchants selling products with “helping farmers” attributes on Taobao, Jingdong, and some farming assistance mini-programs, as well as disclosing supply chain information.

^2^Dragon social reporting in 2020 https://www.dragonsocial.net/blog/social-media-in-china/#WeChat.

^3^TechNode briefing in 2019 https://technode.com/2019/01/11/wechat-founder-1-billion-users/.

## References

[B1] Ackers B. (2015). Ethical considerations of corporate social responsibility-a south african perspective. South Afr. J. Bus. Manag. 46, 11–21. 10.4102/sajbm.v46i1.79

[B2] Alexander R. D. (2017). The Biology of Moral Systems. London: Routledge.

[B3] Amini M. Bienstock C. C. (2014). Corporate sustainability: an integrative definition and framework to evaluate corporate practice and guide academic research. J. Clean Prod. 76, 12–19. 10.1016/j.jclepro.2014.02.016

[B4] Amir D. Jordan M. R. Rand D. G. (2018). An uncertainty management perspective on long-run impacts of adversity: the influence of childhood socioeconomic status on risk, time, and social preferences. J. Exp. Soc. Psychol. 79, 217–226. 10.1016/j.jesp.2018.07.014

[B5] Anderson C. Hildreth J. A. D. Howland L. (2015). Is the desire for status a fundamental human motive? a review of the empirical literature. Psychol. Bull. 141, 574. 10.1037/a0038781 25774679

[B6] Ashforth B. E. Mael F. (1989). Social identity theory and the organization. Acad. Manag. Rev. 14, 20–39. 10.2307/258189

[B7] Bai C. Sarkis J. (2020). A supply chain transparency and sustainability technology appraisal model for blockchain technology. Int. J. Product. Res. 58, 2142–2162. 10.1080/00207543.2019.1708989

[B8] Baker W. E. Bulkley N. (2014). Paying it forward vs. rewarding reputation: mechanisms of generalized reciprocity. Organ. Sci. 25, 1493–1510. 10.1287/orsc.2014.0920

[B9] Baron R. M. Kenny D. A. (1986). The moderator-mediator variable distinction in social psychological research: conceptual, strategic, and statistical considerations. J. Pers. Soc. Psychol. 51, 1173. 10.1037/0022-3514.51.6.1173 3806354

[B10] Bartlett M. Y. DeSteno D. (2006). Gratitude and prosocial behavior: helping when it costs you. Psychol. Sci. 17, 319–325. 10.1111/j.1467-9280.2006.01705.x 16623689

[B11] Bateson M. Nettle D. Roberts G. (2006). Cues of being watched enhance cooperation in a real-world setting. Biol. Lett. 2, 412–414. 10.1098/rsbl.2006.0509 17148417 PMC1686213

[B12] Baumgartner H. Weijters B. (2012). Commentary on “common method bias in marketing: causes, mechanisms, and procedural remedies”. J. Retail. 88, 563–566. 10.1016/j.jretai.2012.10.003

[B13] Bear A. Rand D. G. (2016). Intuition, deliberation, and the evolution of cooperation. Proc. Natl. Acad. Sci. U.S.A. 113, 936–941. 10.1073/pnas.1517780113 26755603 PMC4743833

[B14] Becker A. (2018). An experimental study of voluntary nonprofit accountability and effects on public trust, reputation, perceived quality, and donation behavior. Nonprofit Volunt. Sector Q. 47, 562–582. 10.1177/0899764018756200

[B15] Behr D. (2017). Assessing the use of back translation: the shortcomings of back translation as a quality testing method. Int. J. Soc. Res. Methodol. 20, 573–584. 10.1080/13645579.2016.1252188

[B16] Bekkers R. (2006). “Keeping the faith: origins of confidence in charitable organizations and its consequences for philanthropy,” in NCVO/VSSN Researching the Voluntary Sector Conference. p. 13–14. Available online at: https://www.researchgate.net/publication/466726662

[B17] Berger J. Rosenholtz S. J. Zelditch Jr M. (1980). Status organizing processes. Annu. Rev. Sociol. 6, 479–508. 10.1146/annurev.so.06.080180.002403

[B18] Bhattacharya C. B. Sen S. (2004). Doing better at doing good: when, why, and how consumers respond to corporate social initiatives. Calif. Manag. Rev. 47, 9–24. 10.2307/41166284

[B19] Blader S. L. Chen Y.-R. (2011). What influences how higher-status people respond to lower-status others? effects of procedural fairness, outcome favorability, and concerns about status. Organ. Sci. 22, 1040–1060. 10.1287/orsc.1100.0558

[B20] Bock D. Thomas V. Wolter J. Saenger C. Xu P. (2021). An extended reciprocity cycle of gratitude: how gratitude strengthens existing and initiates new customer relationships. Psychol. Mark. 38, 564–576. 10.1002/mar.21456

[B21] Boyd R. Richerson P. J. (1989). The evolution of indirect reciprocity. Soc. Networks 11, 213–236. 10.1016/0378-8733(89)90003-8

[B22] Brandt H. Sigmund K. (2006). The good, the bad and the discriminator–errors in direct and indirect reciprocity. J. Theor. Biol. 239, 183–194. 10.1016/j.jtbi.2005.08.045 16257417

[B23] Brownlie J. Howson A. (2005). ‘leaps of faith'and mmr: an empirical study of trust. Sociology 39, 221–239. 10.1177/0038038505050536

[B24] Busalim A. H. (2016). Understanding social commerce: a systematic literature review and directions for further research. Int. J. Inf. Manag. 36, 1075–1088. 10.1016/j.ijinfomgt.2016.06.005

[B25] Busser J. A. Shulga L. V. (2019). Involvement in consumer-generated advertising: effects of organizational transparency and brand authenticity on loyalty and trust. Int. J. Contemp. Hospit. Manag. 31, 1763–1784. 10.1108/IJCHM-10-2017-0685

[B26] Carlo G. (2006). “Care-based and altruistically based morality,” in Handbook of Moral Development, eds M. Killen and J. G. Smetana (New York, NY: Psychology Press), 569–598.

[B27] Centobelli P. Cerchione R. Oropallo E. El-Garaihy W. H. Farag T. Al Shehri K. H. (2022). Towards a sustainable development assessment framework to bridge supply chain practices and technologies. Sustain. Dev. 30, 647–663. 10.1002/sd.2262

[B28] Chalub F. A. Santos F. C. Pacheco J. M. (2006). The evolution of norms. J. Theor. Biol. 241, 233–240. 10.1016/j.jtbi.2005.11.028 16388824

[B29] Chan F. K. Thong J. Y. Brown S. A. Venkatesh V. (2021). Service design and citizen satisfaction with e-government services: a multidimensional perspective. Public Adm Rev. 81, 874–894. 10.1111/puar.13308

[B30] Charness G. Rabin M. (2002). Understanding social preferences with simple tests. Q. J. Econ. 117, 817–869. 10.1162/00335530276019390423174385

[B31] Chin W. W. (2010). “How to write up and report pls analyses,” in Handbook of Partial Least Squares (Berlin; Heidelberg: Springer), 655–690. Available online at: https://scholar.google.com/scholar?hl=en&as_sdt=0%2C5&q=How+to+write+up+and+report+pls+analyses&btnG=

[B32] Clarke N. Barnett C. Cloke P. Malpass A. (2007). Globalising the consumer: doing politics in an ethical register. Polit. Geogr. 26, 231–249. 10.1016/j.polgeo.2006.10.009

[B33] Colquitt J. A. Scott B. A. LePine J. A. (2007). Trust, trustworthiness, and trust propensity: a meta-analytic test of their unique relationships with risk taking and job performance. J. Appl. Psychol. 92, 909. 10.1037/0021-9010.92.4.909 17638454

[B34] Condon P. DeSteno D. (2011). Compassion for one reduces punishment for another. J. Exp. Soc. Psychol. 47, 698–701. 10.1016/j.jesp.2010.11.016

[B35] Connelly B. L. Crook T. R. Combs J. G. Ketchen Jr D. J. Aguinis H. (2018). Competence-and integrity-based trust in interorganizational relationships: which matters more? J. Manag. 44, 919–945. 10.1177/0149206315596813

[B36] Cook T. D. Campbell D. T. Shadish W. (2002). Experimental and Quasi-Experimental Designs for Generalized Causal Inference. Boston, MA: Houghton Mifflin.

[B37] Correia A. Kozak M. Kim S. (2018). Luxury shopping orientations of mainland chinese tourists in hong kong: their shopping destination. Tourism Econ. 24, 92–108. 10.1177/1354816617725453

[B38] Correia A. Kozak M. Kim S. (2019). Investigation of luxury values in shopping tourism using a fuzzy-set approach. J. Travel Res. 58, 77–91. 10.1177/0047287517741005

[B39] Critcher C. R. Gilovich T. (2010). Inferring attitudes from mindwandering. Pers. Soc. Psychol. Bull. 36, 1255–1266. 10.1177/0146167210375434 20625177

[B40] Crocker J. Canevello A. (2008). Creating and undermining social support in communal relationships: the role of compassionate and self-image goals. J. Pers. Soc. Psychol. 95, 555. 10.1037/0022-3514.95.3.555 18729694

[B41] Crocker J. Olivier M.-A. Nuer N. (2009). Self-image goals and compassionate goals: costs and benefits. Self Ident. 8, 251–269. 10.1080/15298860802505160 21218194 PMC3017354

[B42] Cronbach L. J. (1951). Coefficient alpha and the internal structure of tests. Psychometrika 16, 297–334. 10.1007/BF02310555

[B43] Cyr D. Head M. Larios H. (2010). Colour appeal in website design within and across cultures: a multi-method evaluation. Int. J. Hum. Comput. Stud. 68, 1–21. 10.1016/j.ijhcs.2009.08.005

[B44] Danz D. Engelmann D. Kübler D. (2022). Do legal standards affect ethical concerns of consumers? Eur. Econ. Rev. 144, 104044. 10.1016/j.euroecorev.2022.104044

[B45] Das T. K. Teng B.-S. (2001). Trust, control, and risk in strategic alliances: an integrated framework. Organ. Stud. 22, 251–283. 10.1177/0170840601222004

[B46] de Morais L. H. L. Pinto D. C. Cruz-Jesus F. (2021). Circular economy engagement: altruism, status, and cultural orientation as drivers for sustainable consumption. Sustain. Product. Consumpt. 27, 523–533. 10.1016/j.spc.2021.01.019

[B47] Deutsch M. (1958). Trust and suspicion. J. Conflict Resolut. 2, 265–279. 10.1177/002200275800200401

[B48] Diallo M. F. Ben Dahmane Mouelhi N. Gadekar M. Schill M. (2021). Csr actions, brand value, and willingness to pay a premium price for luxury brands: does long-term orientation matter? J. Bus. Ethics 169, 241–260. 10.1007/s10551-020-04486-5

[B49] Douglas S. P. Craig C. S. (2007). Collaborative and iterative translation: an alternative approach to back translation. J. Int. Market. 15, 30–43. 10.1509/jimk.15.1.03033826007

[B50] Dowell D. Morrison M. Heffernan T. (2015). The changing importance of affective trust and cognitive trust across the relationship lifecycle: a study of business-to-business relationships. Ind. Market. Manag. 44, 119–130. 10.1016/j.indmarman.2014.10.016

[B51] Dreze X. Nunes J. C. (2009). Feeling superior: the impact of loyalty program structure on consumers' perceptions of status. J. Consum. Res. 35, 890–905. 10.1086/593946

[B52] Duan Y. Hofer C. Aloysius J. A. (2021). Consumers care and firms should too: on the benefits of disclosing supplier monitoring activities. J. Operat. Manag. 67, 360–381. 10.1002/joom.1129

[B53] Dubois D. Rucker D. D. Galinsky A. D. (2012). Super size me: product size as a signal of status. J. Consum. Res. 38, 1047–1062. 10.1086/661890

[B54] Eastman J. K. Goldsmith R. E. Flynn L. R. (1999). Status consumption in consumer behavior: scale development and validation. J. Market. Theory Pract. 7, 41–52. 10.1080/10696679.1999.1150183917350085

[B55] Egels-Zandén N. Hulthén K. Wulff G. (2015). Trade-offs in supply chain transparency: the case of nudie jeans co. J. Clean. Prod. 107, 95–104. 10.1016/j.jclepro.2014.04.074

[B56] Engelmann D. Fischbacher U. (2009). Indirect reciprocity and strategic reputation building in an experimental helping game. Games Econ. Behav. 67, 399–407. 10.1016/j.geb.2008.12.006

[B57] Falk A. Zehnder C. (2007). Discrimination and In-Group Favoritism in a Citywide Trust Experiment. IZA Discussion Paper No. 2765, Zurich IEER Working Paper No. 318.

[B58] Fang Y. Qureshi I. Sun H. McCole P. Ramsey E. Lim K. H. (2014). Trust, satisfaction, and online repurchase intention. Mis. Q. 38, 407-A9. 10.25300/MISQ/2014/38.2.04

[B59] Fehr E. Fischbacher U. (2003). The nature of human altruism. Nature 425, 785–791. 10.1038/nature02043 14574401

[B60] Finel B. I. Lord K. M. (1999). The surprising logic of transparency. Int. Stud. Q. 43, 315–339. 10.1111/0020-8833.00122

[B61] Flynn F. J. Reagans R. E. Amanatullah E. T. Ames D. R. (2006). Helping one's way to the top: self-monitors achieve status by helping others and knowing who helps whom. J. Pers. Soc. Psychol. 91, 1123. 10.1037/0022-3514.91.6.1123 17144769

[B62] Fuller C. M. Simmering M. J. Atinc G. Atinc Y. Babin B. J. (2016). Common methods variance detection in business research. J. Bus. Res. 69, 3192–3198. 10.1016/j.jbusres.2015.12.00835725563

[B63] Galinsky A. D. Magee J. C. Gruenfeld D. H. Whitson J. A. Liljenquist K. A. (2008). Power reduces the press of the situation: implications for creativity, conformity, and dissonance. J. Pers. Soc. Psychol. 95, 1450. 10.1037/a0012633 19025295

[B64] Garcia-Torres S. Albareda L. Rey-Garcia M. Seuring S. (2019). Traceability for sustainability-literature review and conceptual framework. Supply Chain Manag. 24, 85–106. 10.1108/SCM-04-2018-0152

[B65] Garg N. (2019). Misery wants control: The roles of helplessness and choice in the sadness-consumption relationship. Aust. J. Manag. 44, 407–424. 10.1177/0312896219830152

[B66] Gaskin K. (1999). Blurred vision: public trust in charities. Int. J. Nonprofit Volunt. Sector Market. 4, 163–178. 10.1002/nvsm.66

[B67] Gefen D. Straub D. (2000). The relative importance of perceived ease of use in is adoption: a study of e-commerce adoption. J. Assoc. Inf. Syst. 1, 1–30. 10.17705/1jais.00008

[B68] Gefen D. Straub D. (2005). A practical guide to factorial validity using pls-graph: tutorial and annotated example. Commun. Assoc. Inf. Syst. 16, 5. 10.17705/1CAIS.01605

[B69] Gefen D. Straub D. Boudreau M.-C. (2000). Structural equation modeling and regression: Guidelines for research practice. Commun. Assoc. Inf. Syst. 4, 7. 10.17705/1CAIS.00407

[B70] Gerber A. S. Green D. P. (2012). Field Experiments: Design, Analysis, and Interpretation. WW Norton.

[B71] Gharib R. K. Garcia-Perez A. Dibb S. Iskoujina Z. (2019). Trust and reciprocity effect on electronic word-of-mouth in online review communities. J. Enterprise Inf. Manag. 33, 120–138. 10.1108/JEIM-03-2019-0079

[B72] Gilbert P. (2017). Compassion: Concepts, Research and Applications. Taylor & Francis.

[B73] Gilbert P. (2019). Explorations into the nature and function of compassion. Curr. Opin. Psycholo. 28, 108–114. 10.1016/j.copsyc.2018.12.002 30639833

[B74] Goetz J. L. Keltner D. Simon-Thomas E. (2010). Compassion: an evolutionary analysis and empirical review. Psychol. Bull. 136, 351. 10.1037/a0018807 20438142 PMC2864937

[B75] Goh L. (2022). “How agritech is transforming traditional agriculture in emerging markets,” in Breakthrough: The Promise of Frontier Technologies for Sustainable Development. p. 125. Available online at: 10.1007/978-1-4939-0867-7_7

[B76] Gómez-Corona C. (2020). Sensory and consumer research for good: a review on social responsibility. Curr. Opin. Food Sci. 33, 115–123. 10.1016/j.cofs.2020.03.008

[B77] Goodhue D. L. Lewis W. Thompson R. (2012). Does pls have advantages for small sample size or non-normal data? MIS Q. 36, 981–1001. 10.2307/41703490

[B78] Green T. Peloza J. (2011). How does corporate social responsibility create value for consumers? J. Consum. Market. 28, 48–56. 10.1108/0736376111110194932256429

[B79] Grier S. A. Deshpandé R. (2001). Social dimensions of consumer distinctiveness: the influence of social status on group identity and advertising persuasion. J. Market. Res. 38, 216–224. 10.1509/jmkr.38.2.216.18843

[B80] Griskevicius V. Tybur J. M. Van den Bergh B. (2010). Going green to be seen: status, reputation, and conspicuous conservation. J. Pers. Soc. Psychol. 98, 392. 10.1037/a0017346 20175620

[B81] Guinote A. Cotzia I. Sandhu S. Siwa P. (2015). Social status modulates prosocial behavior and egalitarianism in preschool children and adults. Proc. Natl. Acad. Sci. U.S.A. 112, 731–736. 10.1073/pnas.1414550112 25561527 PMC4311842

[B82] Guo Y. Lu Z. Kuang H. Wang C. (2020). Information avoidance behavior on social network sites: Information irrelevance, overload, and the moderating role of time pressure. Int. J. Inf. Manag. 52, 102067. 10.1016/j.ijinfomgt.2020.102067

[B83] Hager M. A. Hedberg E. C. (2016). Institutional trust, sector confidence, and charitable giving. J. Nonprofit Public Sector Market. 28, 164–184. 10.1080/10495142.2015.1011508

[B84] Hair Jr J. F. Hult G. T. M. Ringle C. M. Sarstedt M. (2021). A Primer on Partial Least Squares Structural Equation Modeling (PLS-SEM). Thousand Oaks, CA: SAGE Publications.

[B85] Hair J. F. Black W. C. Babin B. J. (2010). Multivariate Data Analysis: A Global Perspective, Vol. 7. Upper Saddle River, NJ: Pearson.

[B86] Hair J. F. Risher J. J. Sarstedt M. Ringle C. M. (2019). When to use and how to report the results of pls-sem. Eur. Bus. Rev. 31, 2–24. 10.1108/EBR-11-2018-0203

[B87] Hajli N. (2019). The impact of positive valence and negative valence on social commerce purchase intention. Inf. Technol. People 33, 774–791. 10.1108/ITP-02-2018-0099

[B88] Hajli N. Sims J. Zadeh A. H. Richard M.-O. (2017). A social commerce investigation of the role of trust in a social networking site on purchase intentions. J. Bus. Res. 71, 133–141. 10.1016/j.jbusres.2016.10.004

[B89] Han H. Yu J. Kim W. (2019). Environmental corporate social responsibility and the strategy to boost the airline's image and customer loyalty intentions. J. Travel Tourism Market. 36, 371–383. 10.1080/10548408.2018.1557580

[B90] Hardy C. L. Van Vugt M. (2006). Nice guys finish first: The competitive altruism hypothesis. Pers. Soc. Psychol. Bull. 32, 1402–1413. 10.1177/0146167206291006 16963610

[B91] Harrison R. Shaw D. Newholm T. (2005). “The ethical consumer,” in The Ethical Consumer (Thousand Oaks, CA: SAGE Publications Ltd.), 1–280.

[B92] Henseler J. (2018). Partial least squares path modeling: quo vadis? Qual. Quant. 52, 1–8. 10.1007/s11135-018-0689-6 PMC5794891 29416181

[B93] Höddinghaus M. Sondern D. Hertel G. (2021). The automation of leadership functions: would people trust decision algorithms? Comput. Human Behav. 116, 106635. 10.1016/j.chb.2020.106635

[B94] Horowitz L. M. Krasnoperova E. N. Tatar D. G. Hansen M. B. Person E. A. Galvin K. L.. (2001). The way to console may depend on the goal: Experimental studies of social support. J. Exp. Soc. Psychol. 37, 49–61. 10.1006/jesp.2000.1435

[B95] Huang Z. Benyoucef M. (2013). From e-commerce to social commerce: aclose look at design features. Electron. Commer. Res. Appl. 12, 246–259. 10.1016/j.elerap.2012.12.003

[B96] Hulland J. (1999). Use of partial least squares (pls) in strategic management research: a review of four recent studies. Strategic Manag. J. 20, 195–204. 10.1002/(SICI)1097-0266(199902)20:2andlt;195::AID-SMJ13andgt;3.0.CO;2-7

[B97] Ibrahim M. Ribbers P. M. (2009). The impacts of competence-trust and openness-trust on interorganizational systems. Eur. J. Inf. Syst. 18, 223–234. 10.1057/ejis.2009.17

[B98] Idemudia U. (2011). Corporate social responsibility and developing countries: moving the critical csr research agenda in africa forward. Progr. Dev. Stud. 11, 1–18. 10.1177/146499341001100101

[B99] Iglesias O. Markovic S. Bagherzadeh M. Singh J. J. (2020). Co-creation: a key link between corporate social responsibility, customer trust, and customer loyalty. J. Bus. Ethics 163, 151–166. 10.1007/s10551-018-4015-y

[B100] Jaeger A.-K. Weber A. (2020). Can you believe it? the effects of benefit type versus construal level on advertisement credibility and purchase intention for organic food. J. Cleaner Product. 257, 120543. 10.1016/j.jclepro.2020.120543

[B101] Johnson C. M. Tariq A. Baker T. L. (2018). From gucci to green bags: conspicuous consumption as a signal for pro-social behavior. J. Market. Theory Pract. 26, 339–356. 10.1080/10696679.2018.1487769

[B102] Joo S. Miller E. G. Fink J. S. (2019). Consumer evaluations of csr authenticity: development and validation of a multidimensional csr authenticity scale. J. Bus. Res. 98, 236–249. 10.1016/j.jbusres.2019.01.060

[B103] Kafashan S. Sparks A. Griskevicius V. Barclay P. (2014). “Prosocial behavior and social status,” in The Psychology of Social Status (Springer), 139–158. Available online at: https://link.springer.com/chapter/10.1007/978-1-4939-0867-7_7

[B104] Kamboj S. Sarmah B. Gupta S. Dwivedi Y. (2018). Examining branding co-creation in brand communities on social media: Applying the paradigm of stimulus-organism-response. Int. J. Inf. Manag. 39, 169–185. 10.1016/j.ijinfomgt.2017.12.001

[B105] Kanani R. Glavee-Geo R. (2021). Breaking the uncertainty barrier in social commerce: the relevance of seller and customer-based signals. Electron. Commer Res. Appl. 48, 101059. 10.1016/j.elerap.2021.101059

[B106] Kastanakis M. N. Balabanis G. (2012). Between the mass and the class: antecedents of the “bandwagon” luxury consumption behavior. J. Bus. Res. 65, 1399–1407. 10.1016/j.jbusres.2011.10.005

[B107] Katok E. Leider S. Donohue K. (2018). The Handbook of Behavioral Operations. New York, NY: John Wiley & Sons.

[B108] Khan S. Fazili A. I. (2019). Does the need for social status among price conscious consumers induces consumption of counterfeit luxury brands? J. Bus. Manag. 25, 43–70. 10.6347/JBM.201909_25(2).0003

[B109] Kim P. H. Ferrin D. L. Cooper C. D. Dirks K. T. (2004). Removing the shadow of suspicion: the effects of apology versus denial for repairing competence-versus integrity-based trust violations. J. Appl. Psychol. 89, 104. 10.1037/0021-9010.89.1.104 14769123

[B110] Korndörfer M. Egloff B. Schmukle S. C. (2015). A large scale test of the effect of social class on prosocial behavior. PLoS ONE 10, e0133193. 10.1371/journal.pone.0133193 26193099 PMC4507988

[B111] Leitch S. R. (2017). The transparency construct in corporate marketing. Eur. J. Mark. 51, 1503–1509. 10.1108/EJM-07-2017-0456

[B112] Lemay Jr E. P. Clark M. S. (2008). How the head liberates the heart: projection of communal responsiveness guides relationship promotion. J. Pers. Soc. Psychol. 94, 647. 10.1037/0022-3514.94.4.647 18361677

[B113] Li C.-J. Li F. Chen T. Crant J. M. (2022). Proactive personality and promotability: Mediating roles of promotive and prohibitive voice and moderating roles of organizational politics and leader-member exchange. J. Bus. Res. 145, 253–267. 10.1016/j.jbusres.2022.03.002

[B114] Li Y. Wright M. F. (2014). Adolescents' social status goals: relationships to social status insecurity, aggression, and prosocial behavior. J. Youth Adolesc. 43, 146–160. 10.1007/s10964-013-9939-z 23526208

[B115] Liang L. J. Choi H. C. Joppe M. (2018). Exploring the relationship between satisfaction, trust and switching intention, repurchase intention in the context of airbnb. Int. J. Hospit. Manag. 69, 41–48. 10.1016/j.ijhm.2017.10.015

[B116] Lim D. DeSteno D. (2016). Suffering and compassion: the links among adverse life experiences, empathy, compassion, and prosocial behavior. Emotion 16, 175. 10.1037/emo0000144 26751630

[B117] Lindell M. K. Whitney D. J. (2001). Accounting for common method variance in cross-sectional research designs. J. Appl. Psychol. 86, 114. 10.1037/0021-9010.86.1.114 11302223

[B118] Liu M. Wang C. (2010). Explaining the influence of anger and compassion on negotiators' interaction goals: an assessment of trust and distrust as two distinct mediators. Communic Res. 37, 443–472. 10.1177/0093650210362681

[B119] Lonati S. Quiroga B. F. Zehnder C. Antonakis J. (2018). On doing relevant and rigorous experiments: review and recommendations. J. Operat. Manag. 64, 19–40. 10.1016/j.jom.2018.10.003

[B120] Lui S. S. Ngo H.-Y. (2004). The role of trust and contractual safeguards on cooperation in non-equity alliances. J. Manag. 30, 471–485. 10.1016/j.jm.2004.02.002

[B121] Lupoli M. J. Zhang M. Yin Y. Oveis C. (2020). A conflict of values: when perceived compassion decreases trust. J. Exp. Soc. Psychol. 91, 104049. 10.1016/j.jesp.2020.104049

[B122] MacKenzie S. B. Podsakoff P. M. (2012). Common method bias in marketing: causes, mechanisms, and procedural remedies. J. Retail. 88, 542–555. 10.1016/j.jretai.2012.08.001

[B123] MacKenzie S. B. Podsakoff P. M. Podsakoff N. P. (2011). Construct measurement and validation procedures in mis and behavioral research: integrating new and existing techniques. MIS Q. 35, 293–334. 10.2307/23044045

[B124] Mannan M. Ahamed R. Zaman S. B. (2019). Consumers' willingness to purchase online mental health services. J. Serv. Market. 33, 557–571. 10.1108/JSM-05-2018-0163

[B125] Marková I. Marková I. (2004). Trust and Democratic Transition in Post-Communist Europe, Vol. 123. Oxford: Oxford University Press.

[B126] Marshall D. McCarthy L. McGrath P. Harrigan F. (2016). What's your strategy for supply chain disclosure? MIT Sloan Manag. Rev. 57, 37–45. Available online at: https://researchrepository.ucd.ie/server/api/core/bitstre

[B127] Mayer R. C. Davis J. H. Schoorman F. D. (1995). An integrative model of organizational trust. Acad. Manag. Rev. 20, 709–734. 10.2307/258792

[B128] McKnight D. H. Choudhury V. Kacmar C. (2002). Developing and validating trust measures for e-commerce: an integrative typology. Inf. Syst. Res. 13, 334–359. 10.1287/isre.13.3.334.81

[B129] Miret J. García de Vicuña J. L. Guzmán R. Camacho A. Moradi Ghahderijani M. (2017). A flexible experimental laboratory for distributed generation networks based on power inverters. Energies 10, 1589. 10.3390/en10101589

[B130] Mishler W. (2002). The moral foundations of trust. J. Public Policy 22, 353. 10.1017/S0143814X02232043

[B131] Mohr L. A. Webb D. J. Harris K. E. (2001). Do consumers expect companies to be socially responsible? the impact of corporate social responsibility on buying behavior. J. Consum. Affairs 35, 45–72. 10.1111/j.1745-6606.2001.tb00102.x

[B132] Nathoo S. Shaw D. G. Sandy P. T. (2021). Determinants of compassion in providing care to older people: educational implications. Nurse Educ. Today 101, 104878. 10.1016/j.nedt.2021.104878 33798988

[B133] Nicolaou A. I. Ibrahim M. Van Heck E. (2013). Information quality, trust, and risk perceptions in electronic data exchanges. Decis. Support Syst. 54, 986–996. 10.1016/j.dss.2012.10.024

[B134] Nicolaou A. I. McKnight D. H. (2006). Perceived information quality in data exchanges: effects on risk, trust, and intention to use. Inf. Syst. Res. 17, 332–351. 10.1287/isre.1060.0103

[B135] Nitzl C. Roldan J. L. Cepeda G. (2016). Mediation analysis in partial least squares path modeling: helping researchers discuss more sophisticated models. Ind. Manag. Data Syst. 116, 1849–1864. 10.1108/IMDS-07-2015-0302

[B136] Nooteboom B. (2001). Trust: Forms, Foundations, Functions, Failures and Figures. Citeseer.

[B137] Nowak M. A. Roch S. (2007). Upstream reciprocity and the evolution of gratitude. Proc. R. Soc. B Biol. Sci. 274, 605–610. 10.1098/rspb.2006.0125 17254983 PMC2197219

[B138] Nowak M. A. Sigmund K. (1998). Evolution of indirect reciprocity by image scoring. Nature 393, 573–577. 10.1038/31225 9634232

[B139] Nowak M. A. Sigmund K. (2005). Evolution of indirect reciprocity. Nature 437, 1291–1298. 10.1038/nature04131 16251955

[B140] Ohtsuki H. Iwasa Y. (2006). The leading eight: social norms that can maintain cooperation by indirect reciprocity. J. Theor. Biol. 239, 435–444. 10.1016/j.jtbi.2005.08.008 16174521

[B141] Ordabayeva N. Chandon P. (2011). Getting ahead of the joneses: when equality increases conspicuous consumption among bottom-tier consumers. J. Consum. Res. 38, 27–41. 10.1086/658165

[B142] Park J. Chae H. Choi J. N. (2017). The need for status as a hidden motive of knowledge-sharing behavior: an application of costly signaling theory. Human Perform. 30, 21–37. 10.1080/08959285.2016.1263636

[B143] Park S. Tussyadiah I. P. (2020). How guests develop trust in hosts: an investigation of trust formation in p2p accommodation. J. Travel Res. 59, 1402–1412. 10.1177/0047287519884654

[B144] Patzelt H. Shepherd D. A. (2011). Recognizing opportunities for sustainable development. Entrepreneurship Theory Pract. 35, 631–652. 10.1111/j.1540-6520.2010.00386.x

[B145] Pavlou P. A. Fygenson M. (2006). Understanding and predicting electronic commerce adoption: an extension of the theory of planned behavior. MIS Q. 30, 115–143. 10.2307/25148720

[B146] Penner L. A. Dovidio J. F. Piliavin J. A. Schroeder D. A. (2005). Prosocial behavior: multilevel perspectives. Annu. Rev. Psychol. 56, 365–392. 10.1146/annurev.psych.56.091103.070141 15709940

[B147] Pfattheicher S. Nielsen Y. A. Thielmann I. (2022). Prosocial behavior and altruism: a review of concepts and definitions. Curr. Opin. Psychol. 44, 124–129. 10.1016/j.copsyc.2021.08.021 34627110

[B148] Piferi R. L. Lawler K. A. (2006). Social support and ambulatory blood pressure: an examination of both receiving and giving. Int. J. Psychophysiol. 62, 328–336. 10.1016/j.ijpsycho.2006.06.002 16905215

[B149] Piff P. K. Kraus M. W. Côté S. Cheng B. H. Keltner D. (2010). Having less, giving more: the influence of social class on prosocial behavior. J. Pers. Soc. Psychol. 99, 771. 10.1037/a0020092 20649364

[B150] Piff P. K. Robinson A. R. (2017). Social class and prosocial behavior: current evidence, caveats, and questions. Curr. Opin. Psychol. 18, 6–10. 10.1016/j.copsyc.2017.06.003 29221512

[B151] Pinto D. C. Herter M. M. Rossi P. Nique W. M. Borges A. (2019). Recycling cooperation and buying status: Effects of pure and competitive altruism on sustainable behaviors. Eur. J. Market. 53, 944–971. 10.1108/EJM-09-2017-0557

[B152] Podnar K. (2008). Guest editorial: communicating corporate social responsibility. J. Market. Commun. 14, 75–81. 10.1080/13527260701856350

[B153] Podsakoff N. (2003). Common method biases in behavioral research: a critical review of the literature and recommended remedies. J. Appl. Psychol. 885, 10–1037. 10.1037/0021-9010.88.5.879 14516251

[B154] Podsakoff P. M. MacKenzie S. B. Podsakoff N. P. (2012). Sources of method bias in social science research and recommendations on how to control it. Annu. Rev. Psychol. 63, 539–569. 10.1146/annurev-psych-120710-100452 21838546

[B155] Podsakoff P. M. Organ D. W. (1986). Self-reports in organizational research: problems and prospects. J. Manag. 12, 531–544. 10.1177/0149206386012004088452065

[B156] Pommier E. Neff K. D. Tóth-Király I. (2020). The development and validation of the compassion scale. Assessment 27, 21–39. 10.1177/1073191119874108 31516024

[B157] Preacher K. J. Hayes A. F. (2004). Spss and sas procedures for estimating indirect effects in simple mediation models. Behav. Res. Methods Instruments Comput. 36, 717–731. 10.3758/BF03206553 15641418

[B158] Preacher K. J. Hayes A. F. (2008). Asymptotic and resampling strategies for assessing and comparing indirect effects in multiple mediator models. Behav. Res. Methods 40, 879–891. 10.3758/BRM.40.3.879 18697684

[B159] Puska P. Kurki S. Lähdesmäki M. Siltaoja M. Luomala H. (2018). Sweet taste of prosocial status signaling: when eating organic foods makes you happy and hopeful. Appetite 121, 348–359. 10.1016/j.appet.2017.11.102 29180074

[B160] Rand D. G. (2016). Cooperation, fast and slow: meta-analytic evidence for a theory of social heuristics and self-interested deliberation. Psychol. Sci. 27, 1192–1206. 10.1177/0956797616654455 27422875

[B161] Rand D. G. Nowak M. A. (2013). Human cooperation. Trends Cogn. Sci. 17, 413–425. 10.1016/j.tics.2013.06.003 23856025

[B162] Resnick P. Zeckhauser R. Swanson J. Lockwood K. (2006). The value of reputation on ebay: a controlled experiment. Exp. Econ. 9, 79–101. 10.1007/s10683-006-4309-2

[B163] Roberts G. (2008). Evolution of direct and indirect reciprocity. Proc. R. Soc. B Biol. Sci. 275, 173–179. 10.1098/rspb.2007.1134 17971326 PMC2596181

[B164] Roberts G. Raihani N. Bshary R. Manrique H. M. Farina A. Samu F.. (2021). The benefits of being seen to help others: indirect reciprocity and reputation-based partner choice. Philos. Trans. R. Soc. B 376, 20200290. 10.1098/rstb.2020.0290 34601903 PMC8487748

[B165] Rockenbach B. Milinski M. (2006). The efficient interaction of indirect reciprocity and costly punishment. Nature 444, 718–723. 10.1038/nature05229 17151660

[B166] Romano A. Saral A. S. Wu J. (2022). Direct and indirect reciprocity among individuals and groups. Curr. Opin. Psychol. 43, 254–259. 10.1016/j.copsyc.2021.08.003 34481332

[B167] Saberi S. Kouhizadeh M. Sarkis J. Shen L. (2019). Blockchain technology and its relationships to sustainable supply chain management. Int. J. Product. Res. 57, 2117–2135. 10.1080/00207543.2018.1533261

[B168] Sako M. (2006). “Does trust improve business performance,” in Trust Within and Between Organizations: Conceptual Issues and Empirical Applications, eds C. Lane and R. Bachmann (Oxford: Oxford University Press), 88–117.

[B169] Saleh M. A. Ali M. Y. Quazi A. (2013). A comparative study of consumer and b2b goods importers' trust and commitment: evidence from an asian developing country. Aust. Market. J. 21, 126–136. 10.1016/j.ausmj.2012.11.002

[B170] Sammer K. Wüstenhagen R. (2006). The influence of eco-labelling on consumer behaviour-results of a discrete choice analysis for washing machines. Bus. Strategy Environ. 15, 185–199. 10.1002/bse.522

[B171] Samu F. Takács K. (2021). Evaluating mechanisms that could support credible reputations and cooperation: cross-checking and social bonding. Philos. Trans. R. Soc. B 376, 20200302. 10.1098/rstb.2020.0302 34601908 PMC8487741

[B172] Sarkar S. Chauhan S. Khare A. (2020). A meta-analysis of antecedents and consequences of trust in mobile commerce. Int. J. Inf. Manag. 50, 286–301. 10.1016/j.ijinfomgt.2019.08.008

[B173] Saslow L. R. Willer R. Feinberg M. Piff P. K. Clark K. Keltner D.. (2013). My brother's keeper? compassion predicts generosity more among less religious individuals. Soc. Psychol. Pers. Sci. 4, 31–38. 10.1177/1948550612444137

[B174] Schaltegger S. Hansen E. G. Lüdeke-Freund F. (2016). Business models for sustainability: origins, present research, and future avenues. Organ. Environ. 29, 3–10. 10.1177/1086026615599806

[B175] Schaltegger S. Wagner M. (2011). Sustainable entrepreneurship and sustainability innovation: categories and interactions. Bus. Strategy Environ. 20, 222–237. 10.1002/bse.682

[B176] Schmid L. Shati P. Hilbe C. Chatterjee K. (2021). The evolution of indirect reciprocity under action and assessment generosity. Sci. Rep. 11, 1–14. 10.1038/s41598-021-96932-1 34465830 PMC8408181

[B177] Seinen I. Schram A. (2006). Social status and group norms: indirect reciprocity in a repeated helping experiment. Eur. Econ. Rev. 50, 581–602. 10.1016/j.euroecorev.2004.10.005

[B178] Sen S. Du S. Bhattacharya C. (2016). Corporate social responsibility: a consumer psychology perspective. Curr. Opin. Psychol. 10, 70–75. 10.1016/j.copsyc.2015.12.014

[B179] Shao Z. Pan Z. (2019). Building guanxi network in the mobile social platform: a social capital perspective. Int. J. Inf. Manag. 44, 109–120. 10.1016/j.ijinfomgt.2018.10.002

[B180] Shim D. (2021). Capturing heterogeneous decision making processes: the case with the e-book reader market. Int. J. Market Res. 63, 216–235. 10.1177/1470785320980628

[B181] Shrout P. E. Bolger N. (2002). Mediation in experimental and nonexperimental studies: new procedures and recommendations. Psychol. Methods 7, 422. 10.1037/1082-989X.7.4.422 12530702

[B182] Silic M. Ruf C. (2018). The effects of the elaboration likelihood model on initial trust formation in financial advisory services. Int. J. Bank Market. 36, 572–590. 10.1108/IJBM-02-2017-0038

[B183] Silva A. S. Mace R. (2015). Inter-group conflict and cooperation: field experiments before, during and after sectarian riots in northern ireland. Front. Psychol. 6, 1790. 10.3389/fpsyg.2015.01790 26640449 PMC4661283

[B184] Simpson B. Willer R. (2008). Altruism and indirect reciprocity: the interaction of person and situation in prosocial behavior. Soc. Psychol. Q. 71, 37–52. 10.1177/019027250807100106

[B185] Skarmeas D. Leonidou C. N. (2013). When consumers doubt, watch out! the role of csr skepticism. J. Bus. Res. 66, 1831–1838. 10.1016/j.jbusres.2013.02.004

[B186] Sodhi M. S. Tang C. S. (2019). Research opportunities in supply chain transparency. Product. Operat. Manag. 28, 2946–2959. 10.1111/poms.13115

[B187] Sommerfeld R. D. Krambeck H.-J. Semmann D. Milinski M. (2007). Gossip as an alternative for direct observation in games of indirect reciprocity. Proc. Natl. Acad. Sci. U.S.A. 104, 17435–17440. 10.1073/pnas.0704598104 17947384 PMC2077274

[B188] Song H. Wang J. Han H. (2019). Effect of image, satisfaction, trust, love, and respect on loyalty formation for name-brand coffee shops. Int. J. Hospit. Manag. 79, 50–59. 10.1016/j.ijhm.2018.12.011

[B189] Spikins P. (2015). How Compassion Made us Human: The Evolutionary Origins of Tenderness, Trust and Morality. Pen and Sword.

[B190] Stamos A. Lange F. Huang S.-,c. Dewitte S. (2020). Having less, giving more? two preregistered replications of the relationship between social class and prosocial behavior. J. Res. Pers. 84, 103902. 10.1016/j.jrp.2019.103902

[B191] Suzuki S. Kimura H. (2013). Indirect reciprocity is sensitive to costs of information transfer. Sci. Rep. 3, 1–5. 10.1038/srep01435 23486389 PMC3595703

[B192] Szcześniak M. Świątek A. H. Świątek M. A. Rodzeń W. (2020). Positive downstream indirect reciprocity scale (podirs-6): construction and psychometric characteristics. Curr. Psychol. 41, 4379–4400. 10.1007/s12144-020-00942-7

[B193] Taheri F. Shourmasti M. A. (2016). Effects of various characteristics of social commerce on consumers' trust and trust performance. Int. Acad. J. Bus. Manag. 3, 20–26. 10.1016/j.ijinfomgt.2012.11.006

[B194] Tajfel H. Turner J. C. (2004). “The social identity theory of intergroup behavior,” in Political Psychology: Key Readings, eds J. T. Jost and J. Sidanius (New York, NY: Psychology Press), 276–293.

[B195] Tajfel H. Turner J. C. Worchel S. Austin W. G.. (1986). Psychology of Intergroup Relations. Chicago: Nelson-Hall.

[B196] Talwar S. Dhir A. Khalil A. Mohan G. Islam A. N. (2020). Point of adoption and beyond. Initial trust and mobile-payment continuation intention. J. Retail. Consum. Serv. 55, 102086. 10.1016/j.jretconser.2020.102086

[B197] Teo T. S. Liu J. (2007). Consumer trust in e-commerce in the united states, singapore and china. Omega 35, 22–38. 10.1016/j.omega.2005.02.001

[B198] Thielmann I. Böhm R. Ott M. Hilbig B. E. (2021). Economic games: an introduction and guide for research. Collabra Psychol. 7, 19004. 10.1525/collabra.1900432430167

[B199] Trivers R. L. (1971). The evolution of reciprocal altruism. Q. Rev. Biol. 46, 35–57. 10.1086/406755

[B200] Twyman M. Harvey N. Harries C. (2008). Trust in motives, trust in competence: separate factors determining the effectiveness of risk communication. Judgm Decis. Mak. 3, 111. 10.1017/S1930297500000218

[B201] van Dijk E. De Dreu C. K. (2021). Experimental games and social decision making. Annu. Rev. Psychol. 72, 415–438. 10.1146/annurev-psych-081420-110718 33006926

[B202] Vanhamme J. Grobben B. (2009). “too good to be true!”. The effectiveness of csr history in countering negative publicity. J. Bus. Ethics 85, 273–283. 10.1007/s10551-008-9731-2

[B203] Viswanathan M. Kayande U. (2012). Commentary on “common method bias in marketing: causes, mechanisms, and procedural remedies”. J. Retail. 88, 556–562. 10.1016/j.jretai.2012.10.002

[B204] Wang Y. Min Q. Han S. (2016). Understanding the effects of trust and risk on individual behavior toward social media platforms: a meta-analysis of the empirical evidence. Comput. Human Behav. 56, 34–44. 10.1016/j.chb.2015.11.011

[B205] Wedekind C. Milinski M. (2000). Cooperation through image scoring in humans. Science 288, 850–852. 10.1126/science.288.5467.850 10797005

[B206] Weir J. P. (2005). Quantifying test-retest reliability using the intraclass correlation coefficient and the sem. J. Strength Condit. Res. 19, 231–240. 10.1519/00124278-200502000-00038 15705040

[B207] Williams C. C. (2005). Trust diffusion: the effect of interpersonal trust on structure, function, and organizational transparency. Bus. Soc. 44, 357–368. 10.1177/0007650305275299

[B208] Williams L. J. Hartman N. Cavazotte F. (2010). Method variance and marker variables: a review and comprehensive cfa marker technique. Organ. Res. Methods 13, 477–514. 10.1177/1094428110366036

[B209] Wongkitrungrueng A. Assarut N. (2020). The role of live streaming in building consumer trust and engagement with social commerce sellers. J. Bus. Res. 117, 543–556. 10.1016/j.jbusres.2018.08.032

[B210] World Social Report. (2021). Reconsidering Rural Development. United Nations Department of Economic and Social Affairs. Available online at: https://www.un.org/en/desa/world-social-report-2021

[B211] Wu J.-H. Lin L.-M. Rai A. Chen Y.-C. (2022). How health care delivery organizations can exploit ehealth innovations: an integrated absorptive capacity and it governance explanation. Int. J. Inf. Manag. 65, 102508. 10.1016/j.ijinfomgt.2022.102508

[B212] Wu J. B. Tsui A. S. Kinicki A. J. (2010). Consequences of differentiated leadership in groups. Acad. Manag. J. 53, 90–106. 10.5465/amj.2010.48037079

[B213] Wu X. Dluhošová D. Zmeškal Z. (2021). Corporate social responsibility and profitability: the moderating role of firm type in chinese appliance listed companies. Energies 14, 227. 10.3390/en14010227

[B214] Xiao L. Zhang Y. Fu B. (2019). Exploring the moderators and causal process of trust transfer in online-to-offline commerce. J. Bus. Res. 98, 214–226. 10.1016/j.jbusres.2019.01.069

[B215] Xie C. Bagozzi R. P. Grønhaug K. (2015). The role of moral emotions and individual differences in consumer responses to corporate green and non-green actions. J. Acad. Market. Sci. 43, 333–356. 10.1007/s11747-014-0394-5

[B216] Xu Y. Zhang Y. (2022). Institutional trust and prosocial behavior in china: an experimental approach. J. Soc. Psychol. 163, 79–93. 10.1080/00224545.2022.2050881 35311476

[B217] Yang X. (2021). Determinants of consumers' continuance intention to use social recommender systems: a self-regulation perspective. Technol. Soc. 64, 101464. 10.1016/j.techsoc.2020.101464

[B218] Ylitalo J. (2009). Controlling for common method variance with partial least squares path modeling: A monte carlo study. Research project, Helsinki University of Technology.

[B219] Yu W. Han X. Cui F. (2022). Increase consumers' willingness to pay a premium for organic food in restaurants: explore the role of comparative advertising. Front. Psychol. 13, 982311. 10.3389/fpsyg.2022.982311 35992425 PMC9381812

[B220] Zahavi A. (1995). Altruism as a handicap: the limitations of kin selection and reciprocity. J. Avian Biol. 26, 1–3. 10.2307/3677205

[B221] Zhang L. Yang W. Zheng X. (2018). Corporate social responsibility: the effect of need-for-status and fluency on consumers' attitudes. Int. J. Contemporary Hospit. Manag. 30, 1492–1507. 10.1108/IJCHM-01-2017-0048

[B222] Zhao X. Lynch Jr J. G. Chen Q. (2010). Reconsidering baron and kenny: myths and truths about mediation analysis. J. Consum. Res. 37, 197–206. 10.1086/651257

[B223] Zhou L. Wang W. Xu J. D. Liu T. Gu J. (2018). Perceived information transparency in b2c e-commerce: an empirical investigation. Inf. Manag. 55, 912–927. 10.1016/j.im.2018.04.005

[B224] Zhuang M. Zhu W. Huang L. Pan W.-T. (2021). Research of influence mechanism of corporate social responsibility for smart cities on consumers' purchasing intention. Library Hi Tech 40, 1147–1158. 10.1108/LHT-11-2020-0290

